# Cardiovascular Risk Factors and Clinical Outcomes among Patients Hospitalized with COVID-19: Findings from the World Heart Federation COVID-19 Study

**DOI:** 10.5334/gh.1128

**Published:** 2022-06-15

**Authors:** Dorairaj Prabhakaran, Kavita Singh, Dimple Kondal, Lana Raspail, Bishav Mohan, Toru Kato, Nizal Sarrafzadegan, Shamim Hayder Talukder, Shahin Akter, Mohammad Robed Amin, Fastone Goma, Juan Gomez-Mesa, Ntobeko Ntusi, Francisca Inofomoh, Surender Deora, Evgenii Philippov, Alla Svarovskaya, Alexandra Konradi, Aurelio Puentes, Okechukwu S. Ogah, Bojan Stanetic, Aurora Issa, Friedrich Thienemann, Dafsah Juzar, Ezequiel Zaidel, Sana Sheikh, Dike Ojji, Carolyn S. P. Lam, Junbo Ge, Amitava Banerjee, L. Kristin Newby, Antonio Luiz P. Ribeiro, Samuel Gidding, Fausto Pinto, Pablo Perel, Karen Sliwa, On behalf of the WHF COVID-19 Study Collaborators

**Affiliations:** 1 Public Health Foundation India, Centre for Chronic Disease Control, World Heart Federation, London School of Hygiene & Tropical Medicine, GB; 2 Public Health Foundation of India, Gurugram, Haryana, India, and Centre for Chronic Disease Control, New Delhi, IN; 3 Heidelberg Institute of Global Health, University of Heidelberg, Germany; 4 Centre for Chronic Disease Control, New Delhi, IN; 5 World Heart Federation, Geneva, CH; 6 Department of Cardiology, Dayanand Medical College, Ludhiana, Punjab, IN; 7 Department of Clinical Research, National Hospital Organization Tochigi Medical Centre, JP; 8 Department of Cardiovascular Medicine, Dokkyo Medical University School of Medicine, JP; 9 Isfahan Cardiovascular Research Center, Cardiovascular Research Institute, Isfahan University of Medical Sciences, Isfahan, Iran & School of Population and Public Health, University of British Columbia, Vancouver, CA; 10 Kuwait Bangladesh Friendship Government Hospital, BD; 11 National Coordinator, Eminence, Bangladesh; 12 Dhaka Medical College Hospital, BD; 13 Centre for Primary Care Research/Levy Mwanawasa University Teaching Hospital, Lusaka, ZM; 14 Head. Cardiology Service. Fundación Valle del Lili. Cali, CO; 15 Division of Cardiology, Department of Medicine and Cape Heart Institute, Faculty of Health Sciences, University of Cape Town and Groote Schuur Hospital, ZA; 16 Internal Medicine Department, Olabisi Onabanjo University Teaching Hospital, PMB 2001, Sagamu, NG; 17 Department of Cardiology, All India Institute of Medical Sciences, Jodhpur, IN; 18 Ryazan State Medical University, Ryazan emergency hospital, 85 Stroykova street, Ryazan, RU; 19 Cardiology Research Institute, Tomsk National Research Medical Center, Russian Academy of Sciences, RU; 20 Almazov National Medical Research Centre, St.Petersburg, RU; 21 ISSSTE Clínica Hospital de Guanajuato, Cerro del Hormiguero S/N, Maria de la Luz, 36000 Guanajuato, Gto., Mexico, AS; 22 Department of Medicine, College of Medicine, University of Ibadan, and University College Hospital Ibadan, NG; 23 Department of Cardiology, University Clinical Centre of the Republic of Srpska, BA; 24 Instituto Nacional de Cardiologia, Rio de Janeiro, BR; 25 Cape Heart Institute, Department of Medicine, Faculty of Health Sciences, University of Cape Town, South Africa and Department of Internal Medicine, University Hospital Zurich, University of Zurich, CH; 26 National Cardiovascular Center Harapan Kita Hospital, Jakarta, ID; 27 Department Cardiology & Vascular medicine, University of Indonesia, ID; 28 Cardiology department, Sanatorio Güemes, and Pharmacology department, School of Medicine, University of Buenos Aires. Acuña de Figueroa 1228 (1180AAX), Buenos Aires, AR; 29 Department of clinical Research, Tabba Heart Institute. ST-1, block 2, Federal B area, Karachi, PK; 30 Department of Medicine, Faculty of Clinical Sciences, University of Abuja, and University of Abuja Teaching Hospital, NG; 31 National Heart Center Singapore and Duke-National University of Singapore, SG; 32 Department of Cardiology, University Medical Center Groningen, University of Groningen, Groningen, NL; 33 Department of Cardiology, Zhongshan Hospital, Fudan University. Shanghai Institute of Cardiovascular Diseases, Shanghai, CN; 34 University College London, GB; 35 Duke Clinical Research Institute, Duke University School of Medicine, Durham, NC, US; 36 Cardiology Service and Telehealth Center, Hospital das Clínicas, and Department of Internal Medicine, Faculdade de Medicina, Universidade Federal de Minas Gerais, Belo Horizonte, BR; 37 Santa Maria University Hospital, CAML, CCUL, Faculdade de Medicina da Universidade de Lisboa, Lisbon, PT; 38 Department of Non-communicable Disease Epidemiology, London School of Hygiene & Tropical Medicine, World Heart Federation, CH; 39 Cape Heart Institute, Department of Medicine & Cardiology, Groote Schuur Hospital, Faculty of Health Sciences, University of Cape Town, South Africa, World Heart Federation, CH

**Keywords:** COVID-19, mortality, cardiovascular disease

## Abstract

**Background and aims:**

Limited data exist on the cardiovascular manifestations and risk factors in people hospitalized with COVID-19 from low- and middle-income countries. This study aims to describe cardiovascular risk factors, clinical manifestations, and outcomes among patients hospitalized with COVID-19 in low, lower-middle, upper-middle- and high-income countries (LIC, LMIC, UMIC, HIC).

**Methods:**

Through a prospective cohort study, data on demographics and pre-existing conditions at hospital admission, clinical outcomes at hospital discharge (death, major adverse cardiovascular events (MACE), renal failure, neurological events, and pulmonary outcomes), 30-day vital status, and re-hospitalization were collected. Descriptive analyses and multivariable log-binomial regression models, adjusted for age, sex, ethnicity/income groups, and clinical characteristics, were performed.

**Results:**

Forty hospitals from 23 countries recruited 5,313 patients with COVID-19 (LIC = 7.1%, LMIC = 47.5%, UMIC = 19.6%, HIC = 25.7%). Mean age was 57.0 (±16.1) years, male 59.4%, pre-existing conditions included: hypertension 47.3%, diabetes 32.0%, coronary heart disease 10.9%, and heart failure 5.5%. The most frequently reported cardiovascular discharge diagnoses were cardiac arrest (5.5%), acute heart failure (3.8%), and myocardial infarction (1.6%). The rate of in-hospital deaths was 12.9% (N = 683), and post-discharge 30 days deaths was 2.6% (N = 118) (overall death rate 15.1%). The most common causes of death were respiratory failure (39.3%) and sudden cardiac death (20.0%). The predictors of overall mortality included older age (≥60 years), male sex, pre-existing coronary heart disease, renal disease, diabetes, ICU admission, oxygen therapy, and higher respiratory rates (p < 0.001 for each). Compared to Caucasians, Asians, Blacks, and Hispanics had almost 2–4 times higher risk of death. Further, patients from LIC, LMIC, UMIC versus. HIC had 2–3 times increased risk of death.

**Conclusions:**

The LIC, LMIC, and UMIC’s have sparse data on COVID-19. We provide robust evidence on COVID-19 outcomes in these countries. This study can help guide future health care planning for the pandemic globally.

## Introduction

The coronavirus disease 2019 (COVID-19) pandemic has presented an unprecedented global challenge to health care communities. The pandemic continues to affect the lives of millions worldwide, with substantially growing numbers of infections and deaths in all countries [1,2,3]. Although a large proportion of patients (~80%) with COVID-19 have mild to moderate symptoms, the case-fatality rate is highly variable [4,5,6]. The mortality rate among adults with COVID-19 ranges between 2–7% overall, and up to 20% among the elderly [7,8]. Prior studies suggested that people with established cardiovascular disease (CVD), or those at high CVD risk, develop a more severe course of COVID-19 needing admission to an intensive care unit (ICU) [9] and experience higher mortality [10,11]. Furthermore, recent reports suggest an excess of cardiovascular complications among COVID-19 patients, including acute coronary events, myocardial injury, and heart failure [12].

However, most published reports (~95%) on the epidemiology and management of COVID-19 patients are from high-income countries (HICs) [13]. There is limited documentation from low-, lower-middle-, and upper-middle- income countries (LIC, LMIC, and UMIC) where >90% of the poorest billion in their most productive age-group live [14,15]. Also, it is unclear whether the commonly prevalent CVDs in LICs such as rheumatic heart disease, congenital heart disease, and peripartum and other cardiomyopathies, increase the risk of severe course of COVID-19, leading to poor outcomes. Many LMICs have a high burden of CVD and its risk factors that are associated with greater morbidity and mortality following a COVID-19. Managing seriously ill patients with COVID-19 requires vast resources (medical supplies, ventilators, etc.), emerging as a daunting challenge even in HICs. To plan credible policies to combat the COVID-19 pandemic, a better understanding of how resource-constrained countries are dealing with the COVID-19 pandemic is imperative.

This World Heart Federation (WHF) global study aimed to describe cardiovascular risk factors and clinical outcomes among patients hospitalized with COVID-19 across diverse populations to inform clinical and policy practices.

## Methods

### Study design and setting

**Figure 1:**
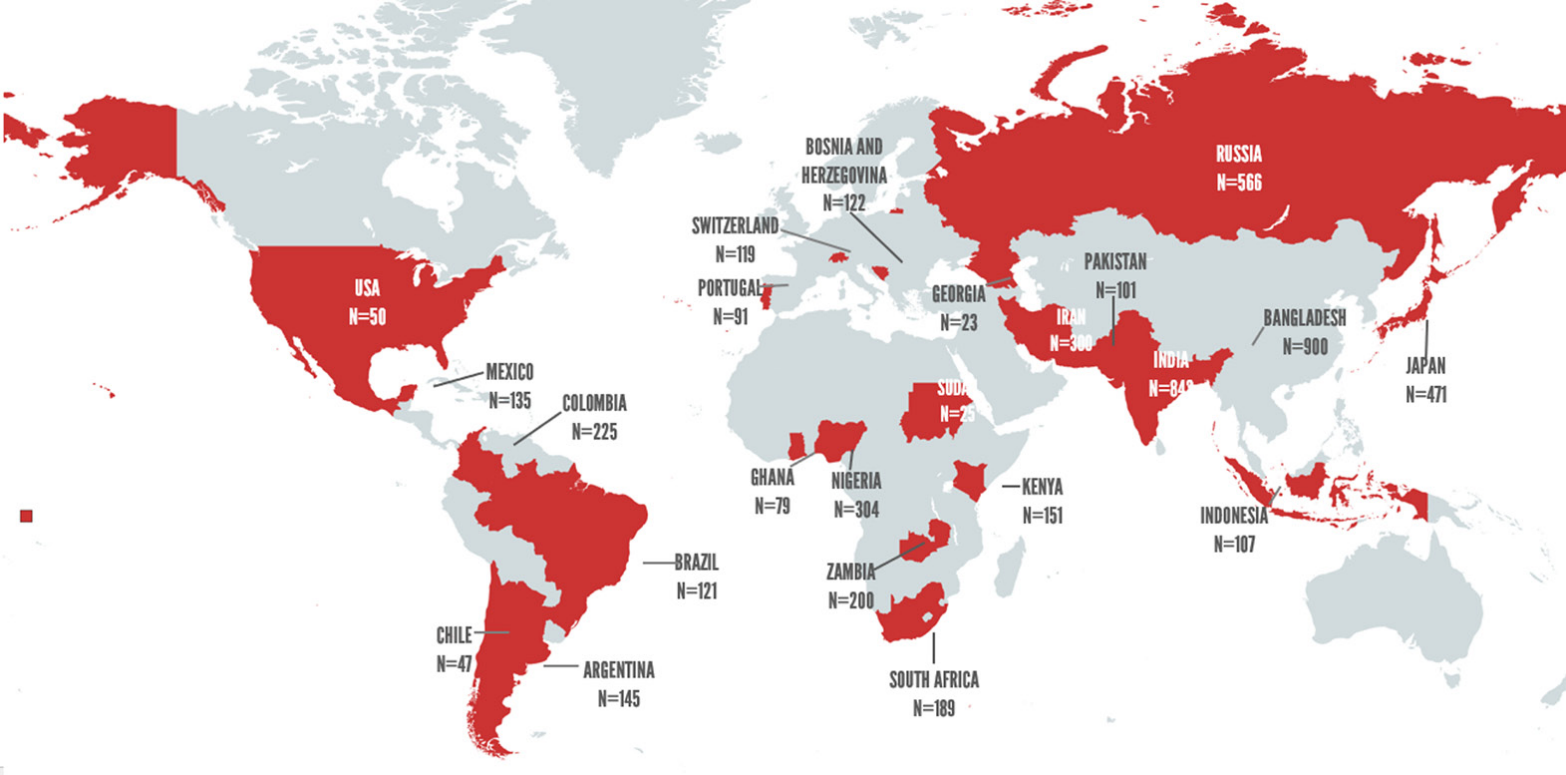
Participating countries and patient recruitment in the WHF COVID-19 study. N = number of patients recruited in the study by participating country.

The full details of the study design, patient recruitment, eligibility criteria, and assessments were reported previously [13]. Briefly, we conducted a prospective cohort study of adults with COVID-19 from hospitals in LIC, LMIC, UMIC, and HIC [16], with a 30-day post-admission follow-up. We invited all WHF members from 100+ countries to identify three recruiting centres in their respective countries. Each centre was expected to recruit between 50 and 500 consecutive COVID-19 patients, depending on the size of the country. Forty hospitals from 23 countries participated in this study. Figure 1 shows the location of the participating countries.

### Study Population

All adults aged 18 years or older with a confirmed reverse transcriptase-polymerase chain reaction (RT-PCR) positive COVID-19 infection who were hospitalized and consented to participate were recruited. Patients who were unlikely to stay in the recruiting centre (i.e., likely to be transferred) or unlikely to be available for a 30 day follow up were excluded.

### Data collection

Hospital level resources and facility data were gathered from each participating hospital. Each hospital provided the following information at the beginning of the study: estimated size of population served, total number of beds, number of intensive care unit (ICU) beds, number of ventilators, number of cardiologists, availability of echocardiogram (ECG) and advanced care interventional and diagnostic capability (e.g., extracorporeal membrane oxygenation [ECMO], echocardiography [ECHO]), and number of COVID-19 patients admitted in the previous month.

Detailed information at the patient level was obtained: demographics (age, sex, ethnicity, education, smoking status and pregnancy status) and clinical characteristics (COVID-19 symptoms and admission vital signs [symptom onset, temperature, oxygen, respiratory rate, blood pressure, height, weight, waist circumference, shortness of breath], co-morbidities before admission [cardiovascular and non-cardiovascular]), pre-hospitalization medications, laboratory tests on admission, other examinations during hospitalization (e.g., ECG, ECHO, Chest-X ray), medications, and supportive care during hospitalization. Clinical outcomes were collected at discharge. All patients were followed until 30 days to determine whether the patient was alive or died (with cause) and whether the patient had any re-hospitalization. Further, ECGs (scanned copies of ECG and/or digital files) and ECHO images from studies conducted as part of the usual clinical care of patients were collected. ECG (XML or image files) and ECHO images were anonymized at sites via provided software and sent via encrypted cloud for central reading (supplement – study CRFs). ECG exams were uploaded to a web-based platform to be read and codified in a centralized reading centre [17] according to the Minnesota Code by experienced and certified cardiologists. Automatic measurements of ECG intervals, including the QT interval, were reviewed.

Study data were collected and managed using the electronic data capture platform REDCap hosted at the Public Health Foundation of India (PHFI) [18,19].

### Outcomes

Study outcomes at hospital discharge included the need for ICUs admission, need for a ventilator, death (with cause), major adverse cardiovascular events (MACE; myocarditis, arrhythmia, heart failure including left ventricular ejection fraction, acute coronary event including cardiac arrest, and acute heart failure), neurological outcomes (ischemic stroke, transient ischemic attack, haemorrhagic stroke), acute renal failure and pulmonary outcomes such as pneumonia, and acute respiratory distress syndrome. In addition, post-discharge death and rehospitalization up to 30-days were collected.

### Sample size and analysis

With a sample size of ≥5200 eligible COVID-19 hospitalized patients, and assuming an incidence of 8% of an adverse outcome such as all-cause mortality and cardiovascular events, this study had more than 90% power to detect adverse outcomes (mortality, MACE) with 95% confidence and a margin of error of 2% (confidence interval). Further, with a sample of ≥5000 COVID-19 patients, and assuming a proportion of potential cardiovascular risk factors such as hypertension or diabetes at 20%, the study has more than 80% power to detect a relative risk of 2.0 for poor outcomes (death and MACE).

### Statistical analyses

The hospital-level resources and facilities available are reported as numbers (percentages), and by WHO region and World Bank income groups. We report overall demographics, clinical characteristics, medication use (pre-admission), laboratory parameters of the study participants (and by status as survivors), in-hospital deaths, and post-discharge 30-day deaths. Data are reported as a number (percentage) for categorical variables, mean (SD) for normally distributed continuous variables, and median (IQR) for skewed distributions. The overall p-value for differences between survivors, in-hospital deaths, and post-discharge 30-day deaths for categorical variables was assessed using the chi-square test and one-way ANOVA for continuous variables. Similarly, the non-fatal clinical outcomes at discharge are reported overall and by status as survivors, in-hospital deaths, and post-discharge 30-day deaths. The p values for differences are also reported.

The association of demographics, other clinical characteristics, and medication use with mortality was assessed using log-binomial regression [20,21]. Unadjusted and adjusted relative risk with 95% confidence intervals were reported. Three regression models were constituted. In Model 1 (demographics), each of the covariates reported was adjusted for age, sex, and ethnicity. In Model 2 (demographics + clinical factors), each of the covariates reported was adjusted for age, sex, ethnicity, history of diabetes, and history of asthma or COPD. In Model 3 (demographics + clinical factors + smoking), each of the covariates reported was adjusted for age, sex, ethnicity, history of diabetes, history of asthma or COPD, and smoking. In case of convergence issues with log-binomial regression, the Poisson regression model [22] was applied, and relative risk with 95% confidence interval was reported.

An exploratory analysis was performed using multinomial logistics regression to assess the association of demographics, clinical characteristics, and medication use with outcome, i.e., in-hospital deaths and post-discharge 30-day deaths. Both unadjusted and adjusted relative risk with 95% confidence intervals were reported. Similar models were used for the primary outcome analyses, i.e., overall mortality vs survivors. All analyses were performed using the Stata 16.0 MP version.

### Ethical considerations

Institutional ethics approval for the project was obtained from the University of Cape Town, South Africa, and the coordinating centres in India (PHFI and Centre for Chronic Disease Control, New Delhi, India). Additionally, all participating site investigators obtained ethical approval from their respective institutional ethics committees prior to patient recruitment in the study. Mandated national regulatory clearances were also obtained. Patients who voluntarily agreed to participate in the study gave informed consent.

## Results

Forty hospitals (LIC = 4, LMIC = 1 5, UMIC = 8, HIC = 13) from 23 countries (Figure 1) recruited 5,313 COVID-19 patients (LIC = 7.1%, LMIC = 47.5%, UMIC = 19.6%, HIC = 25.7%) who were enrolled between 06 June 2020 – 15 September 2021, and >98% participants completed their 30-day follow-up. The site wise patient recruitment is provided in the Supplement – Appendix 1.

Tables 1a and 1b present the overall hospital level resources/facilities, and by the WHO regions, and the World Bank income groups, respectively. Nearly half of the participating sites were University teaching hospitals, 21% were community or district level hospitals, and 13% were private clinics. Specialist care, ICU/ventilation, and advanced care comprising ECHO and Cath Lab were available across all regions, except sub-optimal specialists and advanced care reported in sites from South Asia (LMIC’s) and Africa (LIC’s).

**Table 1a:** Hospital level resources and facilities available at the participating sites (Overall).


SPECIALISTS	N (%)

*Respiratory consultants (N = 34)*	31 (91%)

*Infectious disease specialists (N = 34)*	28 (82%)

*Cardiologists (N = 35)*	34 (97%)

**Equipment’s for advanced care**	

*ICU (N = 39)*	39 (100%)

*Ventilators (N = 39)*	39 (100%)

*No. of ventilators, mean (SD) (N = 33)*	40.4 (44.8)

*ECG (N = 39)*	39 (100%)

*ECHO (N = 39)*	38 (97%)

*ECMO (N = 37)*	22 (59%)

*Cardiac Cath lab (N = 39)*	33 (85%)

**COVID-19 admissions in the last month, mean (SD)** (N = 35)	275.1 (561.9)

**Administrative status of hospital** (N = 38)	

*Community/District Hospital*	8 (21%)

*University Hospital*	18 (47%)

*Private clinic*	5 (13%)


* Analysis excludes missing values. N = total number of hospitals (denominator); n = hospitals with available resources (numerator); SD = standard deviation; ICU = intensive care unit; ECG = electrocardiogram; ECHO = echocardiogram; ECMO = extracorporeal membrane oxygenation.

**Table 1b:** Hospital level resources and facilities by WHO region and World Bank income groups.


WHO REGION (MODIFIED) (NO. OF SITES)	SPECIALISTS^	ICU/VENTILATORS	ADVANCED CARE*

North America (n = 1)	100%	100%	100%

Europe (n = 9)	100%	100%	100%

Western Pacific (n = 3)	100%	100%	100%

Middle East (n = 1)	100%	100%	100%

Latin America (n = 7)	100%	100%	100%

Southeast Asia (n = 10)	83%	100%	95%

Africa (n = 9)	71%	100%	67%

**WORLD BANK INCOME GROUPS (NO. OF SITES)**	**SPECIALISTS^**	**ICU/VENTILATORS**	ADVANCED CARE*

LIC (n = 4)	42%	100%	75%

LMIC (n = 15)	89%	100%	82%

UMIC (n = 8)	100%	100%	100%

HIC (n = 13)	100%	100%	100%


^ Respiratory, Infectious disease, Cardiologists; * ECHO, Cath lab; ICU = intensive care unit. LIC = low-income countries; LMIC = low- and middle-income countries; UMIC = upper-middle income countries; HIC = high-income countries.

Table 2a shows the demographic and clinical characteristics of study participants overall, and according to vital status at discharge and 30 days. A total of 683 (12.9%) individuals died in hospital, and 118 (2.6%) died post-discharge up to 30-days follow-up. Overall, mean age (SD) was 57.0 (16.1) years, 59.4% were male, and 46.0%, 15.1%, 15.0%, 10.2% were identified as Asians, Caucasians, Blacks, and Hispanics, respectively. Overall, 7% of the participants were current smokers, but the smoking status was unknown for 21% of participants. The mean BMI was 26.9 (5.3) Kg/m^2^, and one-quarter of the participants were overweight (BMI 25–29) and 15.6% were obese (BMI ≥ 30 Kg/m^2^).

**Table 2a:** Demographic and clinical characteristics of study participants.


		SURVIVORS	IN-HOSPITAL DEATHS N (%)	POST DISCHARGE 30-DAY DEATHS, N (%)	P-VALUE FOR DIFFERENCE

	N	N (%)			

**N**	5313	4512 (84.9)	683 (12.9)	118 (2.6)	

Age, mean (SD)	57.0 (16.1)	55.6 (16.0)	64.8 (14.2)	65.4 (13.4)	<0.001

Male	3159 (59.4)	2642 (83.6)	431 (13.6)	86 (2.7)	<0.001

Female	2154 (40.5)	1870 (86.8)	252 (11.7)	32 (1.5)	

**Ethnic Origin**					<0.001

Caucasian	800 (15.1)	749 (93.6)	45 (5.6)	6 (0.8)	

Hispanic	542 (10.2)	403 (74.4)	134 (24.7)	5 (0.9)	

Black	796 (15.0)	669 (84)	117 (14.7)	10 (1.3)	

Middle Eastern	315 (5.9)	283 (89.8)	18 (5.7)	14 (4.4)	

Asian	2442 (46.0)	2046 (83.8)	324 (13.3)	72 (2.9)	

Other	346 (6.5)	303 (84.4)	45 (12.5)	11 (3.1)	

**World Bank income groups**					<0.001

LIC	376 (7.1)	331 (88)	39 (10.4)	6 (1.6)	

LMIC	2526 (47.5)	2141 (81.3)	403 (15.3)	89 (3.4)	

UMIC	1044 (19.6)	742 (79.2)	181 (19.3)	14 (1.5)	

HIC	1367 (25.7)	1298 (95)	60 (4.4)	9 (0.7)	

**Education**					<0.001

Up to primary	510 (9.6)	388 (76.1)	110 (21.6)	12 (2.4)	

Up to secondary	1162 (21.9)	1011 (87)	123 (10.6)	28 (2.4)	

College/University	1264 (23.8)	1140 (90.2)	111 (8.8)	13 (1.0)	

Unknown	2291 (43.1)	1906 (82.5)	338 (14.6)	65 (2.8)	

**Smoking status**					<0.001

Never	3080 (58.0)	2664 (86.5)	359 (11.7)	56 (1.8)	

Current	370 (7.0)	342 (92.2)	22 (5.9)	7 (1.9)	

Former	751 (14.1)	645 (85.9)	89 (11.9)	17 (2.3)	

Unknown	1110 (20.9)	861 (77.5)	212 (19.1)	38 (3.4)	

**Body mass index (Kg/m^2^), mean (SD)**	26.9 (5.3)				0.35

Underweight (<18)	71 (1.3)	65 (91.5)	5 (7.0)	1 (1.4)	

Normal weight (18–24)	1414 (26.6)	1246 (87.9)	147 (10.4)	25 (1.8)	0.57

Overweight (25–29)	1289 (24.3)	1137 (88.3)	139 (10.8)	12 (0.9)	

Obese (≥30)	831 (15.6)	730 (88.2)	88 (10.6)	10 (1.2)	


SD = standard deviation.

Table 2b describes the COVID-19 symptoms, and co-morbidities among study participants overall and according to vital status at discharge and 30 days. Overall, almost all hospitalized patients (95%) were diagnosed for COVID-19 by RT-PCR. The median time from symptom onset to admission was 5 (IQR: 3 to 8) days. The most common presenting symptom was cough (68.2%), followed by fever or chills (66.4%), and dyspnoea (62.3%). A history of cardiovascular disease was reported in 32.2% – the most common of which were coronary artery disease (10.9%), heart failure (5.5%), stroke (3.7%) and arrhythmia (3.0%). In terms of cardiovascular risk factors, 47.3% had hypertension and 32.0% had diabetes mellitus. Non-survivors more often presented with significantly higher heart rate, lower diastolic blood pressures, shortness of breath and more frequently had hypertension, diabetes, coronary heart disease, atrial fibrillation, rheumatic heart disease, Chagas disease, valvular disease, and chronic kidney disease (Table 2b).

**Table 2b:** COVID-19 symptoms and comorbidities among study participants.


COVID-SYMPTOMS AND VITAL SIGNS	OVERALL	SURVIVORS	IN-HOSPITAL DEATHS N (%)	POST DISCHARGE 30-DAY DEATHS N (%)

	N (%)	N (%)		

Diagnosed by using RT-PCR	5050 (95.0)	4299(85.0)	644(13.0)	107(2.0)

Median time from symptom onset to admission (IQR) in minutes	5 (3–8)	5 (3–8)	5 (3–8)	4 (2–7)

History of self-reported fever	3526 (66.4)	3002 (85.0)	459 (13)	65 (2)

Cough	3624 (68.2)	3087 (85.0)	472 (13)	65 (2)

Dyspnoea OR Tachypnoea	3308 (62.3)	2689 (81.0)	534(16.0)	85 (3)

Heart rate (beats/min), mean (SD)	92.1 (17.8)	91.2 (17.0)	96.9 (21.6)	95.7 (17.3)

Bradycardia (HR < 60 bpm) mean (SD)	128.8 (20.9)	85 (84)	15 (15)	1 (1)

Tachycardia (HR > 100 bpm) mean (SD)	78.2 (13.0)	1103 (78)	265 (19)	41 (3)

Systolic BP (mmHg), mean (SD)	1341 (25.2)	128.7 (19.9)	129.7 (25.4)	129.7 (26.3)

Diastolic BP (mmHg), mean (SD)	1341 (25.2)	78.5 (12.5)	76.4 (15.4)	77.0 (14.9)

Shortness of Breath (SOB)	1335 (25.1)			

No	479 (9.0)	1251(93)	66 (5)	24 (2)

SOB < 100 m	225 (4.2)	1047(78)	252 (19)	37 (3)

SOB 100–500 m	5050 (95.0)	364(76)	96 (20)	19 (4)

SOB > 500 m	5 (3–8)	203(90)	15 (7)	7 (3)

**Co-morbidities (Cardiovascular)**				

Hypertension	2511 (47.3)	2060 (82)	398 (16)	53 (2)

Diabetes	1700 (32.0)	1346 (79)	306 (18)	48 (3)

Coronary artery disease	580 (10.9)	446 (77)	103 (18)	31 (5)

Heart Failure	290 (5.5)	238 (82)	45 (16)	7 (2)

Stroke	197 (3.7)	159 (81)	28 (14)	10 (5)

Atrial Fibrillation	159 (3.0)	134 (84)	22 (14)	3 (2)

Peripheral vascular disease	106 (2.0)	85 (80)	18 (17)	3 (3)

Cardiomyopathies	60 (1.1)	53 (88)	6 (10)	1 (2)

Rheumatic Heart Disease	56 (1.1)	49 (88)	7 (13)	0 (0)

Chagas disease	36 (0.7)	34 (94)	2 (6)	0 (0)

Congenital heart disease	182 (3.4)	166 (91)	9 (5)	7 (4)

Valvular disease	118 (2.2)	94 (80)	21(18)	3 (3)

**Co-morbidities (Non-Cardiovascular)**				

Chronic kidney disease	404 (7.6)	299 (74)	86 (21)	19 (5)

Chronic pulmonary disease	208 (3.9)	160 (77)	44 (21)	5 (2)

Asthma	219 (4.1)	200 (91)	18 (8)	1 (0)

Chronic Immunosuppression	136 (2.6)	110 (81)	25 (18)	1 (1)

HIV	71 (1.3)	62 (87)	6 (8)	3 (4)

Tuberculosis	56 (1.1)	49 (88)	7 (13)	0 (0)

Cancer on chemotherapy	114 (2.1)	90 (79)	20 (18)	4 (4)

Renal replacement therapy	62 (1.2)	45 (73)	16 (26)	1 (2)

Previous organ transplant	45 (0.8)	38 (84)	7 (16)	0 (0)


Rt-PCR = Reverse Transcription Polymerase Chain Reaction; SD = standard deviation; IQR = Inter quartile range; BP = blood pressure; SOB = Shortness of breath; HIC = high income countries; UMIC = upper middle-income countries; LMIC = lower middle-income countries; LIC = low-income countries; HIV = Human immunodeficiency virus.

Figure 2a shows the comparison of in-hospital deaths and post-discharge 30-day deaths among COVID-19 patients by ethnicity. Compared to Caucasians (6%), Hispanics (25%), Blacks (15%), and Asians (13%) had significantly higher proportions of in-hospital deaths. Furthermore, Middle Eastern (4%), and Asians (3%) had relatively higher post-discharge 30-day deaths than other race/ethnicities (p < 0.001). Figure 2b shows the in-hospital deaths and post-discharge 30-days death by World Bank income groups. Compared to HIC (4%), COVID-19 patients recruited from LIC (10%), LMIC (15%), and UMIC (19%) had significantly higher in-hospital deaths (p < 0.001).

**Figure 2a:**
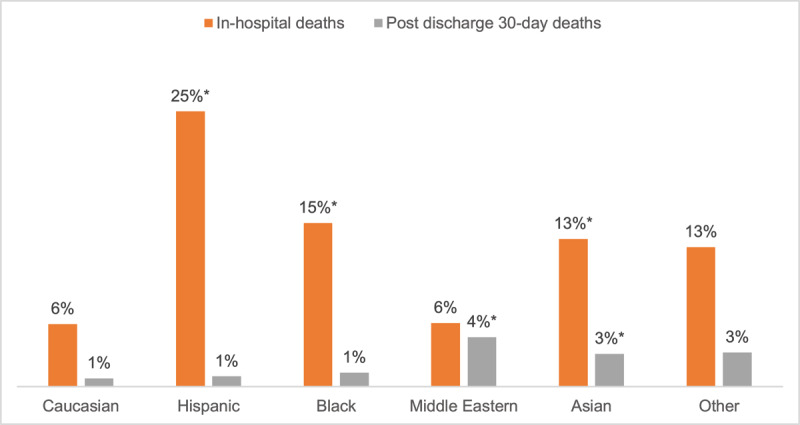
Comparison of in-hospital deaths and post-discharge 30-day deaths among COVID-19 patients by ethnic groups. * p < 0.001 for in-hospital deaths between Caucasians vs. Hispanics, Blacks, and Asians. * P < 0.001 for post-discharge 30-day deaths between Caucasians vs. Middle Eastern and Asians.

**Figure 2b:**
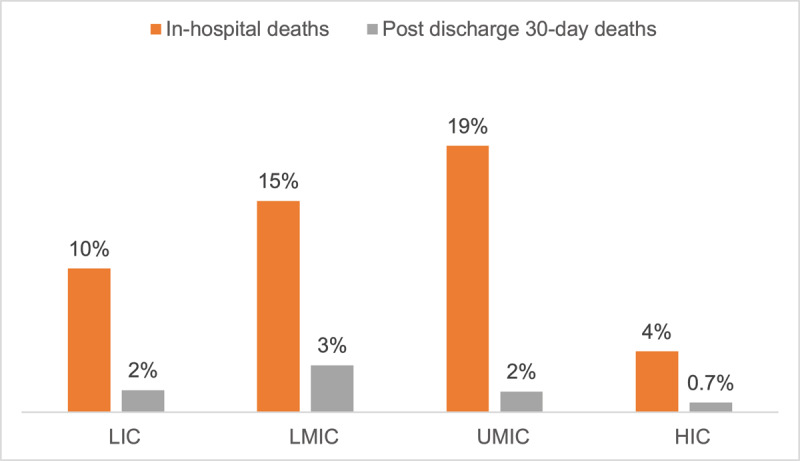
Comparison of in-hospital deaths and post-discharge 30-day deaths in COVID-19 patients by World Bank income groups. * p < 0.001 for both in-hospital deaths and post-discharge 30-day deaths between HIC vs. LIC, LMIC, and UMIC. HIC = high income countries; UMIC = upper middle-income countries; LMIC = lower middle-income countries; LIC = low-income countries.

eTables 1a and 1b shows the demographics and clinical characteristics of study participants, and COVID-19 symptoms/vital signs by World Bank income groups, respectively. Furthermore, a greater number of patients from LMIC (38.9%) and LIC’s (31.7%) reported pre-existing diabetes versus UMIC (20.5%) or HIC (22.0%). eTable 2 shows pre-admission medications. Angiotensin converting enzyme (ACE) inhibitors or angiotensin II receptor blockers were used by 27.1%, beta blockers by 16.9%, diuretics by 11.8%, aspirin by 18.3%, and NSAIDs by 3.5%. Anti-coagulants/antiplatelets, nitrates, diuretics, aldosterone antagonists, oral hypoglycaemic, and insulin were more commonly used by non-survivors than by survivors (p < 0.05 for each). However, NSAIDs and antidepressants were more commonly used by survivors than non-survivors (P < 0.05). Supportive care and medications prescribed during hospitalization are shown in eTable 3. Overall, two-thirds of participants (63.7%) required oxygen therapy, 12.6% required non-invasive ventilation, and 7.4% required invasive ventilation. More than half of the participants were given intravenous fluids (55.8%), corticosteroids (69.5%) and antibiotics (68.1%). Less than a quarter of the participants (23.1%) were prescribed RAAS inhibitors during hospitalization.

Clinical examinations and laboratory results on admission are shown in Table 3. ECG examinations (n = 3497 patients; 65.8%) indicated that 2.5% had atrial fibrillation. The median (IQR) for QT/QTc duration was 419.0 (331.5, 447.0) milli seconds. Nearly 1% of participants who had an echocardiogram (n = 614, 11.6%) had abnormal left ventricular function.

**Table 3:** ECG, ECHO, and laboratory findings among COVID-19 patients at admission.


	OVERALL N (%)	SURVIVORS N (%)	IN-HOSPITAL DEATHS N (%)	POST DISCHARGE 30-DAY DEATHS N (%)	P-VALUE FOR DIFFERENCE

**ECG data (N = 3490)**					

Atrial fibrillation (yes)	131 (2.5)	97 (2.1)	31 (4.5)	3 (2.5)	0.003

T-wave changes (yes)	774 (14.6)	593 (13.1)	153 (22.4)	28 (23.7)	<0.001

QT/QTC duration, median (IQR)	419.0 (331.5, 447.0)	415.5 (259.0, 445.0)	428.0 (360.0, 457.0)	448.0 (413.5, 467.0)	<0.001

**ECHO findings (Median, IQR) (N = 259)**					

Ejection fraction 1. Teicholz (EF1),	59.1 (49.0, 64.0)	60.0 (52.0, 64.0)	55.0 (45.0, 64.0)	59.0 (59.0, 60.0)	0.23

Ejection fraction 2. Visual estimations (EF2),	55.0 (45.0, 60.0)	55.0 (45.0, 60.0)	51.5 (45.0, 59.0)	50.0 (35.0, 55.0)	0.082

**Right ventricular function**					0.002

Mildly/severely abnormal	47 (0.9)	28 (59.1)	18 (38.6)	1 (2.3)	

**Laboratory parameters (median, IQR) (N = 4330)**					

Hemoglobin, mmol/L	7.9 (7.1, 8.8)	8.0 (7.1, 8.8)	7.8 (6.6, 8.7)	7.5 (6.5, 8.4)	<0.001

WBC count, x10^9/L	4.7 (0.0, 8.4)	5.1 (0.0, 8.5)	0.0 (0.0, 7.5)	0.0 (0.0, 6.9)	<0.001

Platelets, 10^3/µL	230.5 (168.0, 336.0)	233.0 (170.0, 342.0)	219.0 (157.0, 306.0)	228.0 (154.0, 425.0)	<0.001

ALT/SGPT, μmol/(s•L)	0.6 (0.4, 1.0)	0.6 (0.4, 1.0)	0.7 (0.4, 1.1)	0.6 (0.4, 1.1)	0.003

AST/SGOT, μmol/(s•L)	0.7 (0.5, 1.1)	0.7 (0.5, 1.0)	0.8 (0.5, 1.4)	0.8 (0.5, 1.3)	<0.001

Creatinine-conversion, μmol/L	87.5 (70.6, 113.2)	85.0 (69.0, 107.0)	99.9 (74.3, 150.3)	104.3 (82.2, 195.4)	<0.001

Sodium, mmol/L	137.0 (134.0, 140.0)	137.0 (134.0, 140.0)	136.3 (133.0, 140.0)	136.0 (133.0, 139.0)	0.10

Potassium, mmol/L	4.2 (3.8, 4.7)	4.2 (3.8, 4.6)	4.3 (3.8, 4.9)	4.5 (4.1, 5.0)	<0.001

CRP, mg/L	53.8 (17.4, 110.7)	48.0 (15.7, 100.0)	93.2 (40.2, 174.0)	82.9 (21.5, 156.1)	<0.001

ESR, mm/hr	43.0 (25.0, 67.0)	41.0 (24.0, 65.0)	52.0 (34.0, 81.0)	53.0 (40.0, 79.0)	<0.001

Troponin, ng/mL	1.0 (0.1, 9.0)	1.0 (0.1, 9.0)	0.1 (0.0, 11.0)	20.0 (2.9, 32.0)	0.007

Troponin T, pg/mL	9.0 (0.5, 24.9)	8.0 (0.6, 20.0)	21.0 (5.5, 64.5)	0.1 (0.0, 16.0)	<0.001

BNP, pmol/L	7.8 (1.5, 28.1)	6.0 (1.2, 21.4)	16.0 (5.1, 49.4)	19.9 (2.2, 44.1)	<0.001

NT-proBNP, pmol/L	60.1 (12.1, 254.4)	46.7 (10.3, 224.2)	110.7 (34.3, 415.5)	505.5 (285.5, 1641.0)	<0.001

CK-Mb, ukat/L,	0.2 (0.0, 13.0)	0.2 (0.0, 13.0)	0.5 (0.0, 19.0)	0.0 (0.0, 0.6)	0.001

Total cholesterol, mmol/L	4.0 (3.1, 5.0)	4.2 (3.4, 5.2)	3.4 (2.7, 4.3)	3.9 (2.5, 4.4)	<0.001

HbA1c,	6.9 (6.1, 8.5)	6.9 (6.1, 8.5)	7.0 (6.2, 8.4)	6.4 (5.9, 9.7)	0.80

D-dimer, mg/FEU/L	1.0 (0.4, 4.4)	0.9 (0.4, 3.9)	1.8 (0.7, 4.8)	2.5 (1.2, 26.5)	<0.001

Ferritin, μg/L	514.1 (225.3, 1001.9)	476.0 (197.5, 962.0)	687.7 (350.3, 1365.2)	656.6 (392.0, 1068.0)	<0.001

IL-6, pg/mL	25.2 (8.7, 64.7)	21.6 (7.0, 52.0)	65.8 (21.9, 125.0)	36.0 (17.6, 133.5)	<0.001

Urea (BUN), mmol/L,	8.5 (5.5, 14.6)	7.7 (5.2, 12.9)	13.9 (7.9, 23.8)	17.0 (10.4, 28.2)	<0.001

PT (seconds)	13.4 (12.0, 15.9)	13.3 (12.0, 15.6)	13.9 (12.1, 16.7)	13.2 (11.7, 16.7)	0.012

INR ratio	1.1 (0.9, 1.3)	1.1 (0.9, 1.2)	1.1 (1.0, 1.3)	1.1 (0.0, 1.3)	0.015


IQR = interquartile range; mmol/L millimoles per liter; mg/L = milligrams per liter.

The incidence of individual cardiovascular events, and other clinical outcomes stratified by survival status at discharge and 30-day follow-up, is presented in Table 4. Overall, the median (IQR) length of hospital stay was 9 (6 to 14) days. About one-third of participants (31.4%) required ICU admission, and the median (IQR) number of days in ICU was 7 (3 to13) days. The most frequently reported clinical outcomes at discharge were pneumonia (37.5%), acute respiratory distress syndrome (13.6%), acute renal injury (8.2%), cardiac arrest (5.5%), shock (5.4%) and acute heart failure (3.8%). Non-survivors more frequently had ICU admission (p < 0.001) and major adverse cardiovascular events, and other adverse clinical outcomes (p < 0.001). The overall vital status at discharge and 30-day follow-up is presented in eTable 4. The most commonly reported causes of death included respiratory failure (39.3%), sudden cardiac death (20.0%) and other cardiovascular deaths (17.4%). A total of 565 participants (16.6%) were not fully recovered from the COVID-19 at 30-day follow-up, and a relatively small proportion of participants (3.8%) responded that their ability to self-care at discharge was worse than before COVID-19.

**Table 4:** Clinical outcomes among COVID-19 patients at discharge.


OUTCOMES AT DISCHARGE	OVERALL N (%)	SURVIVORS N (%)	IN-HOSPITAL DEATHS, N (%)	POST DISCHARGE 30-DAY DEATHS, N (%)	P-VALUE FOR DIFFERENCE

Median length of hospital stays (IQR)	9 (6–14)	9 (6–14)	10 (5–17)	10 (6- 14.5)	0.679

ICU admission	1668 (31.4)	1173 (26.0)	430 (63.0)	65 (55.1)	<0.001

Number of days in ICU (N = 1600), median (IQR)	7 (3–13)	7 (3–11)	9 (4–15)	11 (3–13)	<0.001

Pneumonia	1991 (37.5)	1575 (34.9)	385 (56.4)	31 (26.3)	<0.001

Acute Respiratory Distress Syndrome	723 (13.6)	421 (9.3)	285 (41.7)	17 (14.4)	<0.001

Acute renal injury	436 (8.2)	207 (4.6)	216 (31.6)	13 (11.0)	<0.001

Cardiac arrest	294 (5.5)	56 (1.2)	236 (34.6)	2 (1.7)	<0.001

Anaemia	445 (8.4)	310 (6.9)	123 (18.0)	12 (10.2)	<0.001

Shock	288 (5.4)	52 (1.2)	228 (33.4)	8 (6.8)	<0.001

Acute Heart Failure	200 (3.8)	100 (2.2)	95 (13.9)	5 (4.2)	<0.001

Liver dysfunction	235 (4.4)	172 (3.8)	56 (8.2)	7 (5.9)	<0.001

Atrial Fibrillation	126 (2.4)	86 (1.9)	36 (5.3)	4 (3.4)	<0.001

Pulmonary embolism	116 (2.2)	87 (1.9)	26 (3.8)	3 (2.5)	<0.001

Myocardial Infarction	84 (1.6)	63 (1.4)	18 (2.6)	3 (2.5)	<0.001

Myocarditis	47 (0.9)	22 (0.5)	24 (3.5)	1 (0.8)	<0.001

Ischemic Stroke	56 (1.1)	38 (0.8)	16 (2.3)	2 (1.7)	0.003

Blocks	77 (1.4)	58 (1.3)	19 (2.8)	0 (0.0)	0.033

Ventricular arrhythmia	60 (1.1)	38 (0.8)	21 (3.1)	1 (0.8)	<0.001

Pericarditis	30 (0.6)	27 (0.6)	3 (0.4)	0 (0.0)	0.58

Haemorrhagic Stroke	43 (0.8)	35 (0.8)	8 (1.2)	0 (0.0)	0.046

Endocarditis	14 (0.3)	12 (0.3)	2 (0.3)	0 (0.0)	0.49


IQR = interquartile range; ICU = intensive cardiac unit.

Table 5a shows the factors associated with overall mortality from any cause using the log binomial regression models. As per the final regression Model 3 adjusted for demographics, clinical characteristics and smoking, the elderly (≥60 years) was at three times higher risk of death than younger (<45 years) patients. Male vs. female sex increased the risk of death by at least 16%. Compared to Caucasians, Asians, Blacks, and Hispanics had almost 2–4 times higher risk of death. Likewise, patients recruited from LIC, LMIC, UMIC vs. HIC had 2–3 times increased risk of death. Lastly, pre-existing coronary heart disease, renal disease, diabetes, oxygen therapy use, ICU admission, and higher respiratory rate were all significantly associated with the risk of overall death (p < 0.05 each).

**Table 5a:** Factors associated with overall mortality in COVID-19 hospitalized patients.


	UNADJUSTED RISK	ADJUSTED RISK^1^	ADJUSTED RISK^2^	ADJUSTED RISK^3^
			
	RR (95% CI)	RR (95% CI)	RR (95% CI)	RR (95% CI)

**Age (years)**				

*<45 (Ref)*	1.0	1.0	1.0	1.0

*46–60*	1.92 (1.48, 2.48)	1.91 (1.48, 2.47)	1.81 (1.40, 2.34)	1.78 (1.38, 2.30)

*≥60*	3.59 (2.87, 4.50)	3.76 (3.00, 4.70)	3.45 (2.74, 4.36)	3.40 (2.69, 4.28)

**Sex**				

*Female (Ref)*	1.0	1.0	1.0	1.0

*Male*	1.24 (1.09, 1.42)	1.20 (1.05, 1.36)	1.19 (1.05, 1.36)	1.16 (1.01, 1.33)

**Ethnicity**				

*Caucasian*	1.0	1.0	1.0	1.0

*Hispanic*	4.02 (2.97, 5.44)	4.09 (3.03, 5.51)	3.99 (2.96, 5.38)	3.90 (2.89, 5.25)

*Black*	2.5 (1.84, 3.41)	2.88 (2.12, 3.92)	2.84 (2.09, 3.85)	2.52 (1.87, 3.42)

*Middle Eastern*	1.59 (1.04, 2.43)	1.55 (1.02, 2.36)	1.53 (1.00, 2.32)	1.57 (1.03, 2.39)

*Asian*	2.54 (1.92, 3.37)	2.78 (2.1, 3.67)	2.63 (1.99, 3.48)	2.50 (1.90, 3.30)

*Other*	2.45 (1.71, 3.5)	2.50 (1.75, 3.56)	2.42 (1.70, 3.44)	2.15 (1.51, 3.06)

**Region**				

Europe	1.0	1.0	1.0	1.0

Asia Pacific	0.33 (0.17, 0.67)	0.33 (0.17, 0.67)	0.40 (0.2, 0.79)	0.41 (0.2, 0.81)

Latin America	3.90 (2.9, 5.25)	3.90 (2.9, 5.25)	3.72 (2.77, 5.01)	3.65 (2.71, 4.92)

Middle East	1.62 (1.05, 2.52)	1.62 (1.05, 2.52)	1.54 (0.99, 2.38)	1.60 (1.03, 2.48)

North America	1.04 (0.34, 3.22)	1.04 (0.34, 3.22)	0.96 (0.31, 2.96)	1.02 (0.33, 3.14)

Southeast Asia	3.42 (2.6, 4.51)	3.42 (2.60, 4.51)	3.45 (2.61, 4.56)	3.34 (2.52, 4.42)

Sub Saharan Africa	3.17 (2.36, 4.26)	3.17 (2.36, 4.26)	3.49 (2.61, 4.67)	3.27 (2.44, 4.37)

**Income group**				

*HIC*	1.0		1.0	1.0

*LIC*	2.37 (1.66, 3.39)	2.62 (1.84, 3.73)	2.6 (1.83, 3.71)	2.49 (1.74, 3.57)

*LMIC*	3.62 (2.83, 4.62)	3.55 (2.79, 4.53)	3.42 (2.67, 4.37)	3.22 (2.50, 4.14)

*UMIC*	4.29 (3.32, 5.55)	4.02 (3.11, 5.19)	3.94 (3.04, 5.09)	3.72 (2.86, 4.83)

**Smoking status**				

*Never (Ref)*	1.0	1.0	1.0	1.0

*Current smoker*	0.58 (0.4, 0.83)	0.70 (0.49, 1)	0.71 (0.5, 1.02)	0.71 (0.5, 1.02)

*Former smoker*	1.05 (0.86, 1.28)	0.88 (0.72, 1.08)	0.90 (0.73, 1.10)	0.90 (0.73, 1.10)

*Unknown*	1.67 (1.45, 1.92)	1.46 (1.27, 1.69)	1.48 (1.28, 1.72)	1.48 (1.28, 1.72)

***Pre-existing conditions***				

*Hypertension*	1.40 (1.23, 1.60)	1.07 (0.94, 1.23)	1.01 (0.88, 1.15)	1.03 (0.90, 1.18)

*Coronary heart disease*	1.62 (1.37, 1.90)	1.32 (1.13, 1.55)	1.26 (1.07, 1.49)	1.32 (1.12, 1.57)

*Stroke*	1.29 (0.96, 1.73)	1.09 (0.82, 1.46)	1.08 (0.81, 1.45)	1.14 (0.85, 1.53)

*Heart failure*	1.19 (0.92, 1.53)	1.16 (0.90, 1.48)	1.11 (0.87, 1.43)	1.19 (0.92, 1.53)

*Renal disease*	1.83 (1.53, 2.19)	1.60 (1.35, 1.90)	1.58 (1.32, 1.89)	1.59 (1.33, 1.90)

*COPD/Asthma*	1.02 (0.81, 1.30)	0.96 (0.76, 1.2)	0.96 (0.77, 1.21)	1.00 (0.79, 1.26)

*Diabetes*	1.68 (1.48, 1.91)	1.29 (1.13, 1.46)	1.29 (1.13, 1.46)	1.26 (1.11, 1.44)

*Tuberculosis*	0.82 (0.41, 1.65)	0.78 (0.4, 1.54)	0.80 (0.41, 1.57)	0.83 (0.41, 1.67)

*HIV*	0.84 (0.45, 1.55)	1.05 (0.58, 1.9)	1.08 (0.60, 1.97)	1.18 (0.65, 2.13)

Oxygen therapy	3.02 (2.52, 3.62)	2.59 (2.15, 3.12)	2.54 (2.11, 3.06)	2.53 (2.11, 3.05)

ICU admission	3.48 (3.05, 3.96)	2.91 (2.52, 3.37)	2.96 (2.56, 3.42)	2.84 (2.45, 3.30)

Respiratory rate	1.07 (1.06, 1.07)	1.06 (1.05, 1.07)	1.06 (1.05, 1.07)	1.06 (1.05, 1.07)

**Medications**				

Beta-blockers	0.92 (0.76, 1.11)	0.98 (0.83, 1.15)	0.94 (0.8, 1.11)	0.98 (0.83, 1.16)

Diuretics oral	1.17 (0.96, 1.43)	1.17 (0.98, 1.39)	1.13 (0.95, 1.35)	1.14 (0.95, 1.36)

ACE – inhibitors	0.72 (0.57, 0.91)	0.84 (0.67, 1.06)	0.81 (0.64, 1.02)	0.83 (0.66, 1.04)

Anti-platelets/NOACs	1.35 (1.13, 1.61)	1.19 (1.03, 1.38)	1.14 (0.99, 1.32)	1.18 (1.01, 1.37)

ARBs	1.21 (1.01, 1.45)	1.00 (0.86, 1.17)	0.97 (0.83, 1.13)	1.01 (0.87, 1.18)

Statins	1.03 (0.85, 1.23)	0.91 (0.78, 1.07)	0.85 (0.72, 1.00)	0.88 (0.75, 1.04)

Anti-diabetic drugs	0.69 (0.55, 0.87)	0.96 (0.82, 1.12)	0.71 (0.59, 0.85)	0.74 (0.62, 0.89)

NSAIDs	0.35 (0.2, 0.62)	0.43 (0.25, 0.76)	0.44 (0.25, 0.76)	0.45 (0.26, 0.79)

RAAS inhibitors	0.96 (0.8, 1.15)	0.96 (0.8, 1.15)	0.95 (0.79, 1.14)	0.95 (0.79, 1.14)

**BMI**				

18.0–24.9	1.0	1.0	1.0	1.0

<18.0	0.70 (0.32, 1.52)	1.04 (0.48, 2.25)	1.02 (0.47, 2.2)	1.00 (0.46, 2.17)

25.0–29.9	0.97 (0.79, 1.19)	0.95 (0.78, 1.16)	0.93 (0.77, 1.14)	0.94 (0.77, 1.15)

≥30	0.98 (0.77, 1.23)	1.03 (0.81, 1.30)	0.98 (0.78, 1.24)	0.98 (0.78, 1.24)


RR = relative risk; CI = confidence interval; BMI = body mass index, NOAC = Novel oral anticoagulants; NSAIDs = Nonsteroidal anti-inflammatory drugs; ACE = angiotensin converting enzyme inhibitors’ ARB = angiotensin receptor blockers; RASS = Renin-angiotensin-aldosterone system; ICU = intensive care unit; HIV = human immunodeficiency virus; COPD = chronic obstructive pulmonary disease; HIC = high income countries; UMIC = upper middle-income countries; LMIC = lower middle-income countries; LIC = low-income countries. Adjusted risk^1^ (Model 1): adjusted for demographic variables (age, sex, ethnicity). Adjusted risk^2^ (Model 2): adjusted for demographic and clinical characteristics (Diabetes, COPD/asthma). Adjusted risk^3^ (Model 3): adjusted for demographic and clinical characteristics and smoking.

Table 5b shows the factors associated with MACE in the study population. One thousand nine hundred patients (18.9%) experienced a MACE during their course of admission. Factors associated with MACE adjusted for demographic and clinical characteristics, were older age, male sex, patients recruited from LMICs and UMICs, pre-existing conditions such as hypertension, diabetes, coronary heart disease, stroke, heart failure, and renal disease. Use of oxygen therapy during hospitalization, higher respiratory rate, and ICU admission were significantly associated with increased risk of MACE. On the other hand, current smokers and use of anti-diabetic drugs and patients recruited from LIC’s had significantly (30–40%) lower risk of MACE. These findings were consistent when the multivariable regression model was adjusted for country income groups, in addition to the other demographics and clinical characteristics.

**Table 5b:** Factors associated with major adverse cardiovascular events in COVID-19 hospitalized patients.


	UNADJUSTED RISK	ADJUSTED RISK^1^	ADJUSTED RISK^2^	ADJUSTED RISK^3^
			
	RR (95% CI)	RR (95% CI)	RR (95% CI)	RR (95% CI)

**Age (years)**				

*<45 (Ref)*	1.0	1.0	1.0	1.0

*46–60*	2.06 (1.67, 2.53)	2.03 (1.65, 2.51)	1.91 (1.55, 2.36)	1.90 (1.54, 2.34)

*≥60*	2.94 (2.43, 3.55)	2.90 (2.40, 3.50)	2.63 (2.17, 3.2)	2.59 (2.13, 3.15)

**Sex**				

*Female (Ref)*	1.0	1.0	1.0	1.0

*Male*	1.25 (1.11, 1.41)	1.25 (1.11, 1.40)	1.25 (1.11, 1.4)	1.30 (1.15, 1.47)

**Ethnicity**				

*Caucasian*	1.0	1.0	1.0	1.0

*Hispanic*	1.16 (0.93, 1.44)	1.17 (0.94, 1.45)	1.14 (0.92, 1.41)	1.10 (0.89, 1.36)

*Black*	0.75 (0.6, 0.94)	0.83 (0.66, 1.03)	0.82 (0.65, 1.02)	0.78 (0.62, 0.98)

*Middle Eastern*	1.27 (0.99, 1.63)	1.22 (0.96, 1.56)	1.20 (0.94, 1.53)	1.16 (0.91, 1.48)

*Asian*	1.05 (0.89, 1.24)	1.10 (0.93, 1.30)	1.05 (0.89, 1.23)	1.03 (0.87, 1.21)

*Other*	1.33 (1.05, 1.67)	1.30 (1.04, 1.64)	1.26 (1.00, 1.58)	1.27 (1.01, 1.6)

**Region**				

Asia Pacific	0.07 (0.03, 0.15)	0.08 (0.03, 0.17)	0.08 (0.03, 0.18)	0.08 (0.04, 0.18)

Europe	1.0	1.0	1.0	1.0

Latin America	0.98 (0.79, 1.21)	0.95 (0.77, 1.17)	0.93 (0.75, 1.14)	0.92 (0.74, 1.13)

Middle East	1.27 (0.99, 1.62)	1.21 (0.95, 1.53)	1.19 (0.94, 1.51)	1.17 (0.92, 1.49)

North America	1.39 (0.86, 2.27)	1.35 (0.84, 2.16)	1.27 (0.79, 2.04)	1.25 (0.78, 2.02)

Southeast Asia	1.26 (1.08, 1.48)	1.28 (1.10, 1.50)	1.23 (1.05, 1.44)	1.22 (1.04, 1.42)

Sub Saharan Africa	0.93 (0.77, 1.13)	0.99 (0.82, 1.20)	0.98 (0.81, 1.19)	0.98 (0.81, 1.19)

**Income group**				

*HIC*	1.0	1.0	1.0	1.0

*LIC*	0.69 (0.49, 0.98)	0.73 (0.51, 1.03)	0.72 (0.51, 1.02)	0.68 (0.48, 0.97)

*LMI*C	1.64 (1.40, 1.91)	1.59 (1.36, 1.85)	1.52 (1.30, 1.79)	1.45 (1.23, 1.71)

*UMI*C	1.87 (1.57, 2.22)	1.74 (1.46, 2.07)	1.69 (1.42, 2.01)	1.64 (1.37, 1.95)

**Smoking status**				

*Never (Ref)*	1.0	1.0	1.0	1.0

*Current smoker*	0.61 (0.45, 0.81)	0.59 (0.44, 0.79)	0.60 (0.45, 0.81)	0.60 (0.45, 0.81)

*Former smoker*	1.16 (1.00, 1.36)	0.92 (0.79, 1.08)	0.92 (0.78, 1.08)	0.92 (0.78, 1.08)

*Unknown*	1.11 (0.97, 1.27)	1.00 (0.87, 1.15)	0.98 (0.85, 1.13)	0.98 (0.85, 1.13)

**Pre-existing chronic conditions**				

*Hypertensio*n	1.67 (1.49, 1.87)	1.36 (1.21, 1.53)	1.29 (1.14, 1.45)	1.29 (1.14, 1.45)

*Coronary heart disease*	2.37 (2.11, 2.67)	1.87 (1.64, 2.12)	1.82 (1.6, 2.07)	1.82 (1.60, 2.07)

*Stroke*	1.67 (1.34, 2.07)	1.29 (1.04, 1.6)	1.28 (1.04, 1.59)	1.31 (1.06, 1.62)

*Heart failure*	2.77 (2.43, 3.17)	2.49 (2.16, 2.87)	2.43 (2.11, 2.8)	2.46 (2.13, 2.84)

*Renal disease*	1.92 (1.66, 2.23)	1.68 (1.45, 1.95)	1.62 (1.39, 1.89)	1.62 (1.39, 1.88)

*COPD/Asthma*	1.17 (0.97, 1.42)	1.04 (0.86, 1.25)	1.04 (0.86, 1.26)	1.06 (0.88, 1.28)

*Diabetes*	1.59 (1.43, 1.78)	1.31 (1.17, 1.47)	1.32 (1.17, 1.47)	1.30 (1.16, 1.46)

*Tuberculosis*	0.75 (0.39, 1.42)	0.80 (0.43, 1.51)	0.80 (0.43, 1.5)	0.80 (0.43, 1.50)

*HIV*	0.59 (0.31, 1.14)	0.92 (0.48, 1.78)	0.94 (0.49, 1.82)	0.94 (0.49, 1.82)

Oxygen therapy	2.47 (2.13, 2.87)	2.1 (1.80, 2.46)	2.05 (1.75, 2.40)	2.09 (1.85, 2.35)

ICU admission	2.18 (1.96, 2.43)	2.14 (1.89, 2.42)	2.1 (1.86, 2.36)	2.09 (1.85, 2.35)

Respiratory rate	1.02 (1.02, 1.03)	1.03 (1.02, 1.04)	1.03 (1.02, 1.04)	1.03 (1.02, 1.04)

**Medications**				

Beta-blockers	1.29 (1.12, 1.48)	1.39 (1.23, 1.57)	1.34 (1.18, 1.52)	1.33 (1.18, 1.51)

Diuretics oral	1.44 (1.24, 1.67)	1.57 (1.37, 1.80)	1.53 (1.33, 1.74)	1.54 (1.34, 1.77)

ACE – inhibitors	1.21 (1.03, 1.41)	1.37 (1.17, 1.60)	1.31 (1.12, 1.53)	1.33 (1.13, 1.55)

Anti-coagulant/anti-platelets/NOACs drugs	1.67 (1.45, 1.92)	1.59 (1.41, 1.79)	1.53 (1.35, 1.73)	1.53 (1.35, 1.73)

Angiotensin II receptor blockers	0.89 (0.77, 1.04)	1.00 (0.87, 1.15)	0.97 (0.84, 1.11)	0.98 (0.85, 1.12)

Statins	1.41 (1.23, 1.62)	1.36 (1.2, 1.54)	1.29 (1.14, 1.47)	1.32 (1.16, 1.50)

Anti-diabetic drugs	0.55 (0.46, 0.66)	0.95 (0.83, 1.10)	0.68 (0.58, 0.8)	0.68 (0.58, 0.80)

NSAIDs (regular use)	0.90 (0.68, 1.19)	1.22 (0.93, 1.60)	1.21 (0.93, 1.59)	1.21 (0.92, 1.58)

RASS inhibitors	1.05 (0.91, 1.22)	0.98 (0.84, 1.13)	0.97 (0.84, 1.12)	0.96 (0.83, 1.11)

**BMI (Kg/m^2^)**				

18.0–24.9	1.0	1.0	1.0	1.0

<18.0	0.55 (0.27, 1.13)	0.86 (0.43, 1.73)	0.85 (0.42, 1.71)	0.82 (0.42, 1.61)

25.0–29.9	0.93 (0.79, 1.1)	0.91 (0.77, 1.07)	0.90 (0.76, 1.05)	0.89 (0.75, 1.04)

≥30	1.05 (0.88, 1.26)	1.07 (0.89, 1.29)	1.00 (0.83, 1.20)	1.00 (0.83, 1.21)


BMI = body mass index, NOAC = Novel oral anticoagulants; NSAIDs = Nonsteroidal anti-inflammatory drugs; ACE = angiotensin converting enzyme inhibitors’ ARB = angiotensin receptor blockers; RAAS = Renin-angiotensin-aldosterone system; ICU = intensive care unit; HIV = human immunodeficiency virus; COPD = chronic obstructive pulmonary disease; HIC = high income countries; UMIC = upper middle-income countries; LMIC = lower middle-income countries; LIC = low-income countries. Adjusted risk^1^ (Model 1): adjusted for demographic variables (age, sex, ethnicity). Adjusted risk^2^ (Model 2): adjusted for demographic and clinical characteristics (Diabetes, COPD/asthma). Adjusted risk^3^ (Model 3): adjusted for demographic and clinical characteristics and smoking.

eTable 5 shows the factors associated with in-hospital death and post-discharge 30-day death from any cause, using the multinominal logistic regression models. In addition to older age, male sex, ethnicity (Asian/Middle Eastern) and pre-existing CHD, renal disease, and diabetes, HIV status emerged as a significant factor associated with post-discharge 30-day death but not for in-hospital death.

## Discussion

This study represents the first comprehensive global data on mortality, cardiovascular outcomes, and cardiovascular risk factors among hospitalized COVID-19 patients recruited from diverse global populations. We found a high prevalence of hypertension, diabetes, and cardiovascular disease among patients admitted with COVID-19. The main predictors of mortality or MACE in this WHF study cohort included older age, male sex, pre-existing coronary heart disease, diabetes, renal disease, severe COVID-19 infection with higher respiratory rates and requiring ICU admission and oxygen therapy. We did not find an association of ACE-inhibitors or ARBs with either mortality or cardiovascular events. In the unadjusted regression model, hypertension was significantly associated with mortality, but it did not reach statistical significance in the final model adjusted for both demographic (age, sex, ethnicity, smoking status) and clinical characteristics (diabetes, COPD, asthma). However, hypertension significantly increased the risk of MACE in our cohort. Interestingly, HIV infection was significantly associated with post-discharge 30-day death but not in-hospital death.

Our study is particularly notable for its prospective recruitment of consecutive COVID-19 hospitalized patients across multiple hospital sites spread across LMICs, MICs, and HICs. Further, this is the first global study to explore the relationship of country income status with the clinical outcomes. We found COVID-19 patients recruited from LIC, LMIC and UMIC’s were at significantly greater risk of mortality than HICs. Likewise, patients from LMIC and UMICs vs. HICs had almost 2–3 times increased risk of MACE, but patients from LIC’s had lower risk of MACE, which can be partly explained due to the variation in the demographic characteristics, and pre-existing chronic conditions.

Our analysis demonstrated a greater rate of in-hospital deaths, post discharge 30-day deaths and MACE among Hispanics, and Asian populations compared to Caucasians. Higher prevalence of comorbidities such as hypertension, diabetes, renal disease and obesity among Asians, Hispanics, and other populations (such as Blacks and Middle Eastern populations) may play a role in the increased mortality and MACE in our cohort of COVID-19 patients. The association of ethnicity with in-hospital deaths in patients with COVID-19 has been reported in previous studies [23,24]. Multiple studies from the United States report that Hispanic, Asian or Pacific Islander, and African American patients had higher risk of COVID-19 associated hospitalization and higher mortality compared with White patients after adjusting for sociodemographic factors, and comorbidities [25]. However, disparities in access to health care or potential biases associated with hospital admissions were not considered in many studies, which may influence the rate of COVID-19–related complications and mortality.

Respiratory infections, including previous epidemics of SARS, are known to increase the risk of MACE and mortality [26]. This is particularly well-established for seasonal influenza infections, where vaccination appears to reduce cardiovascular morbidity and mortality by 15% to 20% among high-risk individuals [27,28,29]. Both age and male sex predict adverse outcomes among patients with influenza, and this association remains significant among patients with COVID-19 [28]. However, the exact mechanism for male predominance in the context of COVID-19 infections remains ambiguous. It may be partially explained by sex-related differences in innate and adaptive immunity-links with estrogen receptor signalling [9,10,11]. Further, expression of angiotensin-converting enzyme 2 receptor of SARS-CoV-2 is high in the testes and testosterone, which can cause increased rates of COVID-19 infection among men leading to even higher mortality in critical cases [30,31].

Our findings are also consistent with prior studies that suggested pre-existing CVD was a strong negative prognostic factor [32,33,34,35,36]. Recent studies also showed an excess of cardiovascular complications among COVID-19 patients, such as acute coronary events, myocardial injury, and heart failure [12]. Several systematic reviews and meta-analyses have confirmed that older age, pre-existing CVD, higher troponin T concentration, higher C-reactive protein, and lower albumin are associated with increased mortality among patients hospitalized with COVID-19 [37,38,39,40,41,42]. Indeed, the increased physiological demands imposed by severe infection with COVID-19 may affect people with established CVD more seriously than those without CVD. Poor cardiovascular reserve also unfavourably affects the immune system, potentially predisposing to infection. In our study, although hypertension was significantly associated with mortality in bivariable analysis, it did not emerge as an important risk marker in the adjusted multivariable regression analysis. However, hypertension did significantly increase the risk of MACE in our cohort. A 2020 meta-analysis of 11 studies (all from China) involving 2552 COVID-19 patients reported an OR, 95% CI: of 2.49 (1.98–3.12) for severe disease, and mortality (2.42; 1.51–3.90) in the presence of hypertension [43]. A retrospective cohort study of 1883 COVID-19 patients found two-fold increased risk of COVID-19 mortality in the hypertension vs. non-hypertensive group [44]. An important limitation of the previous literature regarding hypertension and severity of COVID-19 and mortality is the lack of age-adjusted data, and further meta-regression analysis have shown that COVID-19 severity and mortality is seen only in older patients (≥60 years), in whom prevalence of hypertension increases with age.

We found no harmful effects of RAAS inhibitors or NSAIDs. Both classes of drugs received immense attention at the beginning of the COVID-19 pandemic due to their implied role in upregulating the expression of ACE-2, the receptor used by SARS-CoV2 for endocytic internalization [45,46]. Our results are supported by other observational studies of these drug classes and endorse the position statement of the European Society of Cardiology that treatment with ACE inhibitors should not be discontinued in patients with COVID-19. Our study found that HIV status was positively associated with 30-day mortality but not with in-hospital deaths, which suggests a differential immune response like lower likelihood of cytokine storm during acute illness but other susceptibilities coupled with health system factors affecting increased 30-day deaths. Therefore, providing structured and long-term follow-up care for patients with HIV remains an important policy and clinical practice recommendation.

A very low percentage of the patients had received vaccinations at the time of the WHF study being conducted. We are planning to further extend patient recruitment and propose conducting long-term follow-up up to 12 months to study the impact of COVID-19 vaccination, persistence of immunity, and the potential implication of anti-microbial resistance with the severity of COVID-19 illness and adverse outcomes. Further, the long-term follow-up study will address ‘long COVID’ (i.e., long-term downstream clinical consequences and symptoms of COVID-19) as well as aims to elucidate the potential mechanisms/factors contributing to a sizeable proportion of the sudden cardiac deaths observed in our cohort.

### Strengths and Limitations

A major strength of the present study was the global cohort with representation across country income classification (LIC, LMIC, UMIC and HIC). Other important strengths of this study were the geographical coverage (all continents included), consecutive enrolment of hospitalized COVID-19 patients and very high follow-up rate at 30-days (>98%), access to complete medical histories of >95% hospital-screened RT-PCR-diagnosed COVID-19 patients. This allowed us to investigate the importance of pre-existing CVD comorbidities on the absolute risk of severe COVID-19 outcomes in a global cohort of hospitalized COVID-19 patients controlling for age, sex, ethnicity, and other clinical characteristics using multivariate regression analysis.

There are some limitations to our study. As by nature of observational study designs, the results prevented us from making definite inferences regarding causality. It remains uncertain whether the COVID-19 infection is directly involved in the pathogenesis of or acts as a trigger for, cardiovascular events in people with elevated risk. Given the diverse patient population and ethnic groups and multiple comparisons, the univariable and bivariable associations we observed should be interpreted cautiously. Our results apply only to hospitalized patients, and we relied on the routine practice followed at the participating hospital sites for treatment and management of COVID-19, so we did not have comprehensive biomarkers and imaging measurements for all patients recruited in this study.

## Conclusions

This WHF COVID-19 study demonstrated that patients hospitalized with COVID-19 were predominantly middle-aged men with a high prevalence of cardiovascular risk factors such as diabetes and hypertension, and increased mortality at 30 days. The key predictors of mortality were older age (≥ 60 years), male sex, Asian/Hispanic/Black ethnicity, pre-existing coronary heart disease, diabetes, renal disease, severe infection of COVID-19 requiring ICU admission, oxygen therapy and higher respiratory rates, but no significant association was found with hypertension or RAAS inhibitors. Further, the COVID-19 patients from LIC, LMIC, UMIC’s vs. HIC also experienced greater in-hospital mortality, but LIC patients had lower post-discharge 30-day deaths. This study uniquely provides robust evidence on COVID-19 outcomes from LIC, LMIC, and UMICs, which have sparse data on COVID-19 and guides future health care planning for the pandemic globally.

## Additional Files

The Additional files for this article can be found as follows:

10.5334/gh.1128.s1 Supplementary Tables. eTables 1a to 5.

10.5334/gh.1128.s2 Appendix 1. Site wise patient recruitment in the WHF COVID-19 Study.

## Author Notes

Pablo Perel and Karen Sliwa Joint senior authors.

## Research Coordinating Centre: PHFI/CCDC, India

Dorairaj Prabhakaran (Study Co-PI), Kavita Singh (co-investigator, and Senior Research Scientist), Dimple Kondal (Senior Biostatistician), Mumtaj Ali (Senior Data Manager), Mareesha Gandral (Senior Research Assistant), Snehalata Roy (Research Assistant), Sheril Rajan (Analyst).

## Steering Committee

Karen Sliwa (study Co-PI), Dorairaj Prabhakaran (Study Co-PI), Pablo Perel (co-PI), Kavita Singh (co-investigator), Lana Raspail (Project Manager).

## Advisory Committee

K. Srinath Reddy (Chair), Anushka Patel, Barbara Casadei, Cherian Varghese, David Wood, Kay-Tee Khaw, Salim Yusuf.
