# Correction: Cardiovascular Risk Factors and Clinical Outcomes among Patients Hospitalized with COVID-19: Findings from the World Heart Federation COVID-19 Study

**DOI:** 10.5334/gh.1167

**Published:** 2022-10-31

**Authors:** Dorairaj Prabhakaran, Kavita Singh, Dimple Kondal, Lana Raspail, Bishav Mohan, Toru Kato, Nizal Sarrafzadegan, Shamim Hayder Talukder, Shahin Akter, Mohammad Robed Amin, Fastone Goma, Juan Gomez-Mesa, Ntobeko Ntusi, Francisca Inofomoh, Surender Deora, Evgenii Philippov, Alla Svarovskaya, Alexandra Konradi, Aurelio Puentes, Okechukwu S. Ogah, Bojan Stanetic, Aurora Issa, Friedrich Thienemann, Dafsah Juzar, Ezequiel Zaidel, Sana Sheikh, Dike Ojji, Carolyn S. P. Lam, Junbo Ge, Amitava Banerjee, L. Kristin Newby, Antonio Luiz P. Ribeiro, Samuel Gidding, Fausto Pinto, Pablo Perel, Karen Sliwa, On Behalf of the WHF COVID-19 Study Collaborators

**Affiliations:** 1 Public Health Foundation India, Centre for Chronic Disease Control, World Heart Federation, London School of Hygiene & Tropical Medicine, GB; 2 Public Health Foundation of India, Gurugram, Haryana, India, and Centre for Chronic Disease Control, New Delhi, IN; 3 Heidelberg Institute of Global Health, University of Heidelberg, DE; 4 World Heart Federation, Geneva, CH; 5 Department of Cardiology, Dayanand Medical College, Ludhiana, Punjab, IN; 6 Department of Clinical Research, National Hospital Organization Tochigi Medical Centre, JP; 7 Department of Cardiovascular Medicine, Dokkyo Medical University School of Medicine, JP; 8 Isfahan Cardiovascular Research Center, Cardiovascular Research Institute, Isfahan University of Medical Sciences, Isfahan, IR; 9 School of Population and Public Health, University of British Columbia, Vancouver, CA; 10 Kuwait Bangladesh Friendship Government Hospital, BD; 11 National Coordinator, Eminence, BD; 12 Dhaka Medical College Hospital, BD; 13 Centre for Primary Care Research/Levy Mwanawasa University Teaching Hospital, Lusaka, ZM; 14 Head. Cardiology Service. Fundación Valle del Lili. Cali, CO; 15 Division of Cardiology, Department of Medicine and Cape Heart Institute, Faculty of Health Sciences, University of Cape Town and Groote Schuur Hospital, ZA; 16 Internal Medicine Department, Olabisi Onabanjo University Teaching Hospital, PMB 2001, Sagamu, NG; 17 Department of Cardiology, All India Institute of Medical Sciences, Jodhpur, IN; 18 Ryazan State Medical University, Ryazan emergency hospital, 85 Stroykova street, Ryazan, RU; 19 Cardiology Research Institute, Tomsk National Research Medical Center, Russian Academy of Sciences, RU; 20 Almazov National Medical Research Centre, St.Petersburg, RU; 21 ISSSTE Clínica Hospital de Guanajuato, Cerro del Hormiguero S/N, Maria de la Luz, 36000 Guanajuato, Gto., Mexico, AS; 22 Department of Medicine, College of Medicine, University of Ibadan, and University College Hospital Ibadan, NG; 23 Department of Cardiology, University Clinical Centre of the Republic of Srpska, BA; 24 Instituto Nacional de Cardiologia, Rio de Janeiro, BR; 25 Cape Heart Institute, Department of Medicine, Faculty of Health Sciences, University of Cape Town, ZA; 26 Department of Internal Medicine, University Hospital Zurich, University of Zurich, CH; 27 National Cardiovascular Center Harapan Kita Hospital, Jakarta; 28 Department Cardiology & Vascular medicine, University of Indonesia, ID; 29 Cardiology department, Sanatorio Güemes, and Pharmacology department, School of Medicine, University of Buenos Aires. Acuña de Figueroa 1228 (1180AAX), Buenos Aires, AR; 30 Department of clinical Research, Tabba Heart Institute. ST-1, block 2, Federal B area, Karachi, PK; 31 Department of Medicine, Faculty of Clinical Sciences, University of Abuja, and University of Abuja Teaching Hospital, NG; 32 National Heart Center Singapore and Duke-National University of Singapore, SG; 33 Department of Cardiology, University Medical Center Groningen, University of Groningen, Groningen, NL; 34 Department of Cardiology, Zhongshan Hospital, Fudan University. Shanghai Institute of Cardiovascular Diseases, Shanghai, CN; 35 University College London, GB; 36 Duke Clinical Research Institute, Duke University School of Medicine, Durham, NC, US; 37 Cardiology Service and Telehealth Center, Hospital das Clínicas, and Department of Internal Medicine, Faculdade de Medicina, Universidade Federal de Minas Gerais, Belo Horizonte, BR; 38 Santa Maria University Hospital, CAML, CCUL, Faculdade de Medicina da Universidade de Lisboa, Lisbon, PT; 39 Department of Non-communicable Disease Epidemiology, London School of Hygiene & Tropical Medicine, World Heart Federation, CH; 40 Cape Heart Institute, Department of Medicine & Cardiology, Groote Schuur Hospital, Faculty of Health Sciences, University of Cape Town, ZA

**Keywords:** Cardiology, Cardiovascular Health, Global Health, Covid-19

## Abstract

This article details a correction to: Prabhakaran D, Singh K, Kondal D, Raspail L, Mohan B, Kato T, et al. Cardiovascular Risk Factors and Clinical Outcomes among Patients Hospitalized with COVID-19: Findings from the World Heart Federation COVID-19 Study. *Global Heart*. 2022; 17(1): 40. DOI: http://doi.org/10.5334/gh.1128.

## Correction

After the publication of *Cardiovascular Risk Factors and Clinical Outcomes among Patients Hospitalized with COVID-19: Findings from the World Heart Federation COVID-19 Study* [1], the authors noticed some minor errors that required correcting. These fell into four different categories:

1. Some of the tables required amendments.
2. The list of collaborators was incomplete.
3. Some wording throughout the text was not sufficiently precise.
4. The acknowledgement section was incomplete.

### The Table Amendments

One decimal point value was missed in Tables 2a and 2b. 2a was also missing the titles in the first two columns, which were ‘Overall, N (%)’ and ‘Survivors, N(%)’. On Table 3, two decimal point values were added for the blood results: WBC, Troponin T.

**Table 2a:** Demographic and clinical characteristics of study participants.


	OVERALL	SURVIVORS	IN-HOSPITAL DEATHS N (%)	POST DISCHARGE 30-DAY DEATHS, N (%)	P-VALUE FOR DIFFERENCE

	N (%)	N (%)			

**N**	5313	4512 (84.9)	683 (12.9)	118 (2.6)	

Age, mean (SD)	57.0 (16.1)	55.6 (16.0)	64.8 (14.2)	65.4 (13.4)	<0.001

Male	3159 (59.4)	2642 (83.6)	431 (13.6)	86 (2.7)	<0.001

Female	2154 (40.5)	1870 (86.8)	252 (11.7)	32 (1.5)	

**Ethnic Origin**					<0.001

Caucasian	800 (15.1)	749 (93.6)	45 (5.6)	6 (0.8)	

Hispanic	542 (10.2)	403 (74.4)	134 (24.7)	5 (0.9)	

Black	796 (15.0)	669 (84)	117 (14.7)	10 (1.3)	

Middle Eastern	315 (5.9)	283 (89.8)	18 (5.7)	14 (4.4)	

Asian	2442 (46.0)	2046 (83.8)	324 (13.3)	72 (2.9)	

Other	346 (6.5)	303 (84.4)	45 (12.5)	11 (3.1)	

**World Bank income groups**					<0.001

LIC	376 (7.1)	331 (88.0)	39 (10.4)	6 (1.6)	

LMIC	2526 (47.5)	2141 (81.3)	403 (15.3)	89 (3.4)	

UMIC	1044 (19.6)	742 (79.2)	181 (19.3)	14 (1.5)	

HIC	1367 (25.7)	1298 (95)	60 (4.4)	9 (0.7)	

**Education**					<0.001

Up to primary	510 (9.6)	388 (76.1)	110 (21.6)	12 (2.4)	

Up to secondary	1162 (21.9)	1011 (87.0)	123 (10.6)	28 (2.4)	

College/University	1264 (23.8)	1140 (90.2)	111 (8.8)	13 (1.0)	

Unknown	2291 (43.1)	1906 (82.5)	338 (14.6)	65 (2.8)	

**Smoking status**					<0.001

Never	3080 (58.0)	2664 (86.5)	359 (11.7)	56 (1.8)	

Current	370 (7.0)	342 (92.2)	22 (5.9)	7 (1.9)	

Former	751 (14.1)	645 (85.9)	89 (11.9)	17 (2.3)	

Unknown	1110 (20.9)	861 (77.5)	212 (19.1)	38 (3.4)	

**Body mass index (Kg/m^2^), mean (SD)**	26.9 (5.3)				0.35

Underweight (<18)	71 (1.3)	65 (91.5)	5 (7.0)	1 (1.5)	

Normal weight (18–24)	1414 (26.6)	1246 (87.9)	147 (10.4)	25 (1.8)	0.57

Overweight (25–29)	1289 (24.3)	1137 (88.3)	139 (10.8)	12 (0.9)	

Obese (≥30)	831 (15.6)	730 (88.2)	88 (10.6)	10 (1.2)	


SD = standard deviation; row percentage reported for all categorical variables.

**Table 2b:** COVID-19 symptoms and comorbidities among study participants.


COVID-SYMPTOMS AND VITAL SIGNS	OVERALL	SURVIVORS	IN-HOSPITAL DEATHS N (%)	POST DISCHARGE 30-DAY DEATHS N (%)

	N (%)	N (%)		

Diagnosed by using RT-PCR	5050 (95.0)	4299(85.1)	644(12.8)	107(2.1)

Median time from symptom onset to admission (IQR) in minutes	5 (3–8)	5 (3–8)	5 (3–8)	4 (2–7)

History of self-reported fever	3526 (66.4)	3002 (85.1)	459 (13.0)	65 (1.9)

Cough	3624 (68.2)	3087 (85.2)	472 (13.0)	65 (1.8)

Dyspnoea OR Tachypnoea	3308 (62.3)	2689 (81.3)	534(16.1)	85 (2.6)

Heart rate (beats/min), mean (SD)	92.1 (17.8)	91.2 (17.0)	96.9 (21.6)	95.7 (17.3)

Bradycardia (HR<60bpm) mean (SD)	101 (1.9)	85 (84)	15 (15)	1 (1)

Tachycardia (HR>100bpm) mean (SD)	1409 (26.5)	1103 (78)	265 (19)	41 (3)

Systolic BP (mmHg), mean (SD)	128.8 (20.9)	128.7 (19.9)	129.7 (25.4)	129.7 (26.3)

Diastolic BP (mmHg), mean (SD)	78.2 (13.0)	78.5 (12.5)	76.4 (15.4)	77.0 (14.9)

Shortness of Breath (SOB)				

SOB < 100m	1336 (25.5)	1047(78.4)	252 (18.8)	37 (2.8)

SOB 100–500m	479 (9.1)	364(76.0)	96 (20.0)	19 (4.0)

SOB > 500m	225 (4.3)	203(90.2)	15 (6.7)	7 (3.1)

**Co-morbidities (Cardiovascular)**				

Hypertension	2511 (47.3)	2060 (82.0)	398 (16.0)	53 (2.0)

Diabetes	1700 (32.0)	1346 (79.2)	306 (17.8)	48 (3.0)

Coronary artery disease	580 (10.9)	446 (76.9)	103 (17.8)	31 (5.3)

Heart Failure	290 (5.5)	238 (82.1)	45 (15.5)	7 (2.4)

Stroke	197 (3.7)	159 (80.7)	28 (14.2)	10 (5.1)

Atrial Fibrillation	159 (3.0)	134 (84.3)	22 (13.8)	3 (1.9)

Peripheral vascular disease	106 (2.0)	85 (80.2)	18 (17.0)	3 (2.8)

Cardiomyopathies	60 (1.1)	53 (88.3)	6 (10.0)	1 (1.7)

Rheumatic Heart Disease	56 (1.1)	49 (87.5)	7 (12.5)	0 (0)

Chagas disease	36 (0.7)	34 (94.4)	2 (5.6)	0 (0)

Congenital heart disease	182 (3.4)	166 (91.2)	9 (4.9)	7 (3.8)

Valvular disease	118 (2.2)	94 (79.7)	21(17.8)	3 (2.5)

**Co-morbidities (Non-Cardiovascular)**				

Chronic kidney disease	404 (7.6)	299 (74.0)	86 (21.3)	19 (4.7)

Chronic pulmonary disease	208 (3.9)	160 (76.5)	44 (21.1)	5 (2.4)

Asthma	219 (4.1)	200 (91.3)	18 (8.2)	1 (0.5)

Chronic Immunosuppression	136 (2.6)	110 (80.9)	25 (18.4)	1 (0.7)

HIV	71 (1.3)	62 (87.3)	6 (8.5)	3 (4.2)

Tuberculosis	56 (1.1)	49 (87.5)	7 (12.5)	0 (0)

Cancer on chemotherapy	114 (2.1)	90 (78.9)	20 (17.5)	4 (3.6)

Renal replacement therapy	62 (1.2)	45 (72.6)	16 (25.8)	1 (1.6)

Previous organ transplant	45 (0.8)	38 (84.8)	7 (15.6)	0 (0)


Rt-PCR = Reverse Transcription Polymerase Chain Reaction; SD = standard deviation; IQR = Inter quartile range; BP = blood pressure; SOB = Shortness of breath; HIC = high income countries; UMIC = upper middle-income countries; LMIC = lower middle-income countries; LIC = low-income countries; HIV = Human immunodeficiency virus. Row percentage reported for all categorical variables.

**Table 3:** ECG, ECHO, and laboratory findings among COVID-19 patients at admission.


	OVERALL N (%)	SURVIVORS N (%)	IN-HOSPITAL DEATHS N (%)	POST DISCHARGE 30-DAY DEATHS N (%)	P-VALUE FOR DIFFERENCE

**ECG data (N = 3490)**					

Atrial fibrillation (yes)	131 (2.5)	97 (2.1)	31 (4.5)	3 (2.5)	0.003

T-wave changes (yes)	774 (14.6)	593 (13.1)	153 (22.4)	28 (23.7)	<0.001

QT/QTC duration, median (IQR)	419.0 (331.5, 447.0)	415.5 (259.0, 445.0)	428.0 (360.0, 457.0)	448.0 (413.5, 467.0)	<0.001

**ECHO findings (Median, IQR) (N = 259)**					

Ejection fraction 1. Teicholz (EF1),	59.1 (49.0, 64.0)	60.0 (52.0, 64.0)	55.0 (45.0, 64.0)	59.0 (59.0, 60.0)	0.23

Ejection fraction 2. Visual estimations (EF2),	55.0 (45.0, 60.0)	55.0 (45.0, 60.0)	51.5 (45.0, 59.0)	50.0 (35.0, 55.0)	0.082

**Right ventricular function**					0.002

Mildly/severely abnormal	47 (0.9)	28 (59.1)	18 (38.6)	1 (2.3)	

**Laboratory parameters (median, IQR) (N = 4330)**					

Hemoglobin, mmol/L	7.9 (7.1, 8.8)	8.0 (7.1, 8.8)	7.8 (6.6, 8.7)	7.5 (6.5, 8.4)	<0.001

WBC count, ×10^9/L	4.7 (0.0, 8.4)	5.1 (0.0, 8.5)	0.018 (0.009, 7.5)	0.0184 (0.009, 6.9)	<0.001

Platelets, 10^3/µL	230.5 (168.0, 336.0)	233.0 (170.0, 342.0)	219.0 (157.0, 306.0)	228.0 (154.0, 425.0)	<0.001

ALT/SGPT, μmol/(s•L)	0.60 (0.38, 0.97)	0.58 (0.38, 0.95)	0.65 (0.40, 1.11)	0.63 (0.41, 1.09)	0.003

AST/SGOT, μmol/(s•L)	0.67 (0.47, 1.05)	0.65 (0.45, 1.00)	0.79 (0.52, 1.37)	0.82 (0.53, 1.30)	<0.001

Creatinine-conversion, μmol/L	87.5 (70.6, 113.2)	85.0 (69.0, 107.0)	99.9 (74.3, 150.3)	104.3 (82.2, 195.4)	<0.001

Sodium, mmol/L	137.0 (134.0, 140.0)	137.0 (134.0, 140.0)	136.3 (133.0, 140.0)	136.0 (133.0, 139.0)	0.10

Potassium, mmol/L	4.2 (3.8, 4.7)	4.2 (3.8, 4.6)	4.3 (3.8, 4.9)	4.5 (4.1, 5.0)	<0.001

CRP, mg/L	53.8 (17.4, 110.7)	48.0 (15.7, 100.0)	93.2 (40.2, 174.0)	82.9 (21.5, 156.1)	<0.001

ESR, mm/hr	43.0 (25.0, 67.0)	41.0 (24.0, 65.0)	52.0 (34.0, 81.0)	53.0 (40.0, 79.0)	<0.001

Troponin, ng/mL	1.0 (0.1, 9.0)	1.0 (0.1, 9.0)	0.1 (0.037, 11.0)	20.0 (2.9, 32.0)	0.007

Troponin T, pg/mL	9.0 (0.5, 24.9)	8.0 (0.6, 20.0)	21.0 (5.5, 64.5)	0.123 (0.014, 16.0)	<0.001

BNP, pmol/L	7.8 (1.5, 28.1)	6.0 (1.2, 21.4)	16.0 (5.1, 49.4)	19.9 (2.2, 44.1)	<0.001

NT-proBNP, pmol/L	60.1 (12.1, 254.4)	46.7 (10.3, 224.2)	110.7 (34.3, 415.5)	505.5 (285.5, 1641.0)	<0.001

CK-Mb, ukat/L,	0.24 (0.017, 13.0)	0.23 (0.017, 13.0)	0.47 (0.034, 19.0)	0.049 (0.017, 0.613)	0.001

Total cholesterol, mmol/L	4.0 (3.1, 5.0)	4.2 (3.4, 5.2)	3.4 (2.7, 4.3)	3.9 (2.5, 4.4)	<0.001

HbA1c, %	6.9 (6.1, 8.5)	6.9 (6.1, 8.5)	7.0 (6.2, 8.4)	6.4 (5.9, 9.7)	0.80

D-dimer, mg/FEU/L	1.0 (0.4, 4.4)	0.9 (0.4, 3.9)	1.8 (0.7, 4.8)	2.5 (1.2, 26.5)	<0.001

Ferritin, μg/L	514.1 (225.3, 1001.9)	476.0 (197.5, 962.0)	687.7 (350.3, 1365.2)	656.6 (392.0, 1068.0)	<0.001

IL-6, pg/mL	25.2 (8.7, 64.7)	21.6 (7.0, 52.0)	65.8 (21.9, 125.0)	36.0 (17.6, 133.5)	<0.001

Urea (BUN), mmol/L,	8.5 (5.5, 14.6)	7.7 (5.2, 12.9)	13.9 (7.9, 23.8)	17.0 (10.4, 28.2)	<0.001

PT (seconds)	13.4 (12.0, 15.9)	13.3 (12.0, 15.6)	13.9 (12.1, 16.7)	13.2 (11.7, 16.7)	0.012

INR ratio	1.1 (0.9, 1.25)	1.1 (0.9, 1.23)	1.1 (0.96, 1.32)	1.1 (0.0119, 1.32)	0.015


IQR = interquartile range; mmol/L millimoles per liter; mg/L = milligrams per liter.

## The Collaborator List

Four collaborators were inadvertently missed off the original list: L. Tetteh Appiah, Nabil Varwani, Lucky Rose Adika, and Humphrey Robert Guya.

The full, corrected contributor list is:

**Rio de Janeiro, Brazil**, Instituto Nacional de Cardiologia: A. Issa, H. Cramer, C. Lamas, M. Paulino, V. Belidio, L. Sabioni, **Buenos Aires, Argentina**, Hospital de Clinicas of the University: R. Pérez de la Hoz, J. Martin Aladio, M. Matsudo, S. Swieszkowski, A. Perez de la Hoz, **Buenos Aires, Argentina**, Sanatorio Güemes: E. J. Zaidel, J. Perea, M. Ariel Oliva, N. Carboni Martinez, N. Bisso, L. Gheco, **Dhaka, Bangladesh**, Dhaka Medical College Hospital: S. Talukder, S.Akter, M. Robed Amin, M. Ahmedul Kabir, M. Khairul Islam, M. Mohiuddin Sharif, K. Fayzus Salahin, S. Hossain, **Dhaka, Bangladesh**, Kuwait Bangladesh Friendship Govt. Hospital: A. Rahim, K. M. Rubayet Anwar, S. Sajmin Siddiqa, M. Rahman, A. Hossain, **Dhaka, Bangladesh**, Bangladesh Specialized Hospital: A. Wadud Chowdhury, M. Mohiuddin Ahmed, M. Mushfiqur Rahman, U. F. Sultana, Srpska, **Bosnia and Herzegovina**, University Clinical Center Republic of Srpska: B. Stanetic, I. Ovcina, B. Dujakovic, R. Tamburic, D. Vulic, R. Skrbic, **Temuco, Chile**, Hospital Dr. Hernán Henríquez Aravena: P. Figuero, F. La, C. Acs, S. Saavedra Bogota, **Colombia**, Clinica de Occidente: J Lugo-Peña, M. Ángel Zuleta, **Cali**, **Colombia**, Fundacion Valle del Lili: J. Esteban Gomez Mesa, S. Stephania Galindo-Coral, Maria Claudia Montes, **Tbilisi, Georgia**, High Technology Medical Centre: University Clinic (HTMC), K. Chelidze, I. Mamatsashvli, **Kumasi, Ghana**, Komfo Anokye Teaching Hospital: L Tetteh Appiah, Y. Hardy, J. Hutton, **Accra, Ghana**, Military Hospital: A. Toppar, **Ludhiana, India**, Dayanand Medical College Hospital: B. Mohan, M. Mennen, S. Singla, K. Jain, Ankush, **Hyderabad, India**, Apollo Hospital: V. Ram, G. Praveen Kumar, K. Subba Reddy, B. V. K. S. Sowmya, M. Rebecca, **Hyderabad, India**, Apollo Medical College: Jubilee Hills, S. Kuruvada, A. Nimmagadda, A. Begum, **Jodhpur, India**, P. Bhardwaj, J. Charan, S. Deora, D. Sharma, **New Delhi, India**, All India Institute Of Medical Sciences (AIIMS): N Naik, N Rai **Jakarta, Indonesia**, National Cardiovascular Center harapan Kita Hospital: D. Juzar, I Firdaus, B. Putra, M. Rayhan, **Isfahan, Iran**, Amin Hospital: Ladan Sadeghian, N. Sarrafzadegan, Khorshid hospital, **Isfahan, Iran**, Mohammad Hashemi, **Kyoto, Japan**, Kyoto Medical Center: K. Hasegawa, Y. Iida, **Tokyo, Japan**, Kitasato University School of Medicine: J. Ako, R. Kameda, **Tochigi, Japan**, NHO Tochigi Medical Center: T. Kato, **Mombasa, Kenya**, Coast General Teaching and Referral Hospital: E. Ogola, K. Mwazo, V. Vaghela, S. Mohamed, A. Abeid, V. Mumbo, M. Ali Mohamed, A. Ikbal Varvani, M. Omar, V. Karegi, B. Nduati, Swaleh, E. Gacheri, D. Anyanga, S. M. Mohamed, E. Gacheri Riungu, D. Anyanga, Nabil Varwani, **Mombasa, Kenya**, The Mombasa Hospital: S. Mohamed, E. Gacheri Riungu, D. Anyanga, J. Kamuyu Muriuki, K. Rose, Lucky Rose Adika, Humphrey Robert Guya, **Guanajuato, Mexico**, ISSSTE Clínica Hospital de Guanajuato: A. Puentes Puentes, **Lagos Nigeria**, College of Medicine University of Lagos, A. Mbakwem, **Ibadan, Nigeria**, University College Hospital: O. Ogah, O. Adekanmbi, O. Adebayo, Y. Oyebisi, O. Makinde, O.A. Orimolade, O. Makinde, S. Alabi, **Sagamu, Nigeria**, Olabisi Onabanjo University Teaching Hospital: F. Inofomoh, Ranti Familoni, Abimbola Olaitan, Victor Ayeni, Boluwatife Egbetola, **Sindh, Pakistan**, Tabba Heart Institute: S. Sheikh, H. Khan, Z Ahmed, S.F. Ali, R. Malik, **Lisbon, Portugal**, University Hospital Sta Maria: F. Pinto, D. Caldeira, S. Braz, J. Agostinho, J. Brito, H. Barbacena, F. Parlato, C. Carreiro, R. Soares, C. Gomes, A. Pinto Sousa, M. José Pires, **St. Petersburg, Russia**, Almazov National Medical Research Centre: A. Konradi, Z. Kobalava, Y. Yudina, M. Ionov, S. Verbilo, Y. Lavrishcheva, S. Bondar, Y. Khruleva **Moscow, Russia, RUDN University**, City clinical hospital named Vinogradov: L. Contselidze, Y. Khruleva, **Kazan, Russia**, Kazan Clinical Hospital, A. Galyavich, Z. Kim, **Tomsk, Russia**, Tomsk National Research Medical Centre, Asinovskaya Regional Hospital: A. Svarovskaya, A. Kuznetsova, **Ryazan, Russia**, Ryazan State Medical University, Ryazan Emergency Hospital: E. Philippov, **Cape Town, South Africa**, Groote Schuur Hospital: N. A. B. Ntusi, L. Chinhoyi, O. Briton, C. Viljoen, K. Sliwa, P. Singh, S. Mazondwa, M. Mennen, N. Williams, **Khartoum, Sudan**, Fedail Hospital: A. Suliman, **Zurich, Switzerland**, University Hospital of Zurich Hospital: F. Thienemann, V. Rossi, T. Studer, **Atlanta, United States**, A. Quyyumi, M. Prasad, D. Braun, **Lusaka, Zambia**, Levy Mwanawasa University Hospital: F. Goma, N. Mumba. ISSSTE Clínica Hospital de Guanajuato: J. E. Luna Cárdenas, G. Sánchez Loza.

### Necessary Rewording for Clarity

Throughout the article, some sentences required rewording:

1. In the *‘Data Collection’* section:

Each hospital provided the following information at the beginning of the study: estimated size of population served, total number of beds, number of intensive care unit (ICU) beds, number of ventilators, number of cardiologists, availability of echocardiogram (ECG) and advanced care interventional and diagnostic capability (e.g., extracorporeal membrane oxygenation [ECMO], echocardiography [ECHO]), and number of COVID-19 patients admitted in the previous month.

Becomes:

Each hospital provided the following information at the beginning of the study: estimated size of population served, total number of beds, number of intensive care unit (ICU) beds, number of ventilators, number of specialists, availability of echocardiogram (ECG) and advanced care interventional and diagnostic capability (e.g., extracorporeal membrane oxygenation [ECMO], echocardiography [ECHO]), and number of COVID-19 patients admitted in the previous month.

2. In ‘Ethical Considerations’ the following sentence was added: ‘Mandated national regulatory clearances were also obtained.’
3. In ‘Results’:

Non-survivors more often presented with significantly higher heart rate, lower diastolic blood pressures, shortness of breath and more frequently had hypertension, diabetes, coronary heart disease, atrial fibrillation, rheumatic heart disease, Chagas disease, valvular disease, and chronic kidney disease (Table 2b).

Becomes:

Non-survivors more often presented with significantly higher heart rate, lower diastolic blood pressures, shortness of breath and more frequently had hypertension, diabetes, coronary artery disease, stroke, chronic kidney disease, chronic pulmonary disease, asthma and renal replacement therapy. (Table 2b).

4. Further on in the same section, ‘ECG examinations (n = 3497 patients; 65.8%) indicated that 2.5% had atrial fibrillation’ was corrected to ‘ECG examinations (n = 3490 patients; 65.8%) indicated that 2.5% had atrial fibrillation’.
5. In the ‘Discussion’ section, the following passage was reworded from:

Our analysis demonstrated a greater rate of in-hospital deaths, post discharge 30-day deaths and MACE among Hispanics, and Asian populations compared to Caucasians. Higher prevalence of comorbidities such as hypertension, diabetes, renal disease and obesity among Asians, Hispanics, and other populations (such as Blacks and Middle Eastern populations) may play a role in the increased mortality and MACE in our cohort of COVID-19 patients.

To:

Our analysis demonstrated a greater rate of in-hospital deaths, and post discharge 30-day deaths among Hispanics, Asian, Blacks and Middle Eastern populations compared to Caucasians. Higher prevalence of comorbidities such as hypertension, diabetes, renal disease and obesity among Asians, Hispanics, Blacks and Middle Eastern populations may play a role in the increased mortality and MACE in our cohort of COVID-19 patients.

6. In ‘Conclusions’, the following sentence: ‘The key predictors of mortality or MACE outcomes were older age (≥60 years), male sex, Asian/Hispanic/Black ethnicity, pre-existing coronary heart disease, diabetes, renal disease, severe infection of COVID-19 requiring ICU admission, oxygen therapy and higher respiratory rates, but no significant association was found with hypertension or RAAS inhibitors.’ Was corrected by removing ‘or MACE outcomes’.
7. In the ‘Steering Committee’ section, Karen Sliwa (study Co-PI), Dorairaj Prabhakaran (Study Co-PI), Pablo Perel (co-PI), should all have had the same role title of ‘Study Co-PI’.
