# Supplementary material for: Cardiovascular Risk Factors and Clinical Outcomes among Patients Hospitalized with COVID-19: Findings from the World Heart Federation COVID-19 Study
Source: Glob Heart. 2022 Jun 15;17(1):40. doi: 10.5334/gh.1128 (PMC9205371; doi:10.5334/gh.1128)
Supplement: Supplementary Tables. — eTables 1a to 5. [file gh-17-1-1128-s1.pdf]

eTable 1a. Demographic and clinical characteristics of study participants by country income groups (World Bank classification)

	Overall N (%)	HIC N (%)	UMIC N (%)	LMIC N (%)	LIC N (%)	p-value for difference
**N**	**5313**	**1367**	**376**	**2633**	**937**	
Age, mean (SD)	57.0 (16.1)	57.5 (17.5)	53.0 (14.4)	56.6 (15.6)	59.2 (15.5)	<0.001
Male	3158 (59.4%)	762 (55.7%)	232 (61.7%)	1585 (60.2%)	580 (61.9%)	0.006
**Ethnic Origin**						
Caucasian	800 (15.1%)	661 (48.4%)	5 (1.3%)	0 (0.0%)	134 (14.3%)	<0.001
Hispanic	542 (10.2%)	61 (4.5%)	1 (0.3%)	10 (0.4%)	470 (50.2%)	
Black	796 (15.0%)	41 (3.0%)	342 (91.0%)	371 (14.1%)	42 (4.5%)	
Middle Eastern	315 (5.9%)	8 (0.6%)	4 (1.1%)	301 (11.4%)	2 (0.2%)	
Asian	2442 (46.0%)	477 (34.9%)	21 (5.6%)	1942 (73.8%)	2 (0.2%)	
Other	346 (6.5%)	119 (8.7%)	3 (0.8%)	9 (0.3%)	228 (24.3%)	
**WHO Region**						<0.001
Asia Pacific	471 (8.9%)	471 (34.5%)	0 (0.0%)	0 (0.0%)	0 (0.0%)	
Europe	921 (17.3%)	799 (58.4%)	0 (0.0%)	0 (0.0%)	122 (13.0%)	
Latin America	673 (12.7%)	47 (3.4%)	0 (0.0%)	0 (0.0%)	626 (66.8%)	
Middle East	300 (5.6%)	0 (0.0%)	0 (0.0%)	300 (11.4%)	0 (0.0%)	
North America	50 (0.9%)	50 (3.7%)	0 (0.0%)	0 (0.0%)	0 (0.0%)	
Southeast Asia	1950 (36.7%)	0 (0.0%)	0 (0.0%)	1950 (74.1%)	0 (0.0%)	
Africa	948 (17.8%)	0 (0.0%)	376 (100.0%)	383 (14.5%)	189 (20.2%)	
**Education**						<0.001
Up to primary	510 (9.6%)	15 (1.1%)	22 (5.9%)	375 (14.2%)	98 (10.5%)	
Up to secondary	1162 (21.9%)	224 (16.4%)	78 (20.7%)	632 (24.0%)	228 (24.3%)	
College/University	1264 (23.8%)	168 (12.3%)	193 (51.3%)	808 (30.7%)	95 (10.1%)	
Unknown	2291 (43.1%)	960 (70.2%)	83 (22.1%)	818 (31.1%)	448 (47.8%)	
**Smoking status**						<0.001
Never	3080 (58.0%)	695 (50.8%)	274 (72.9%)	1668 (63.3%)	442 (47.2%)	
Current	370 (7.0%)	230 (16.8%)	10 (2.7%)	66 (2.5%)	65 (6.9%)	
Former	751 (14.1%)	269 (19.7%)	30 (8.0%)	257 (9.8%)	195 (20.8%)	
Unknown	1110 (20.9%)	173 (12.7%)	62 (16.5%)	642 (24.4%)	234 (25.0%)	
**Body mass index (Kg/m^2)**						<0.001
Normal weight (18-24)	1414 (26.6%)	482 (35.3%)	46 (12.2%)	787 (29.9%)	103 (11.0%)	
Underweight (<18)	71 (1.3%)	20 (1.5%)	7 (1.9%)	41 (1.6%)	3 (0.3%)	
Overweight (25-29)	1289 (24.3%)	398 (29.1%)	63 (16.8%)	664 (25.2%)	163 (17.4%)	
Obese (>=30)	831 (15.6%)	334 (24.4%)	78 (20.7%)	251 (9.5%)	165 (17.6%)	
**Outcomes**						
Survivors	4512 (84.9%)	1298 (95.0%)	331 (88.0%)	2065 (81.7%)	818 (78.4%)	<0.001
In-hospital deaths	683 (12.9%)	60 (4.4%)	39 (10.4%)	373 (14.8%)	211 (20.2%)	<0.001
Post-discharge 30-day deaths	118 (2.2%)	9 (0.7%)	6 (1.6%)	88 (3.5%)	15 (1.4%)	<0.001
Overall deaths	801 (15.1%)	69 (5.0%)	45 (12.0%)	461 (18.3%)	226 (21.6%)	<0.001
Cardiovascular events	1010 (19.0%)	179 (13.1%)	34 (9.0%)	542 (21.5%)	255 (24.4%)	<0.001

HIC=high income countries; UMIC=upper middle-income countries; LMIC=lower middle-income countries; LIC=low-income countries

eTable 1b. COVID-19 symptoms and comorbidities among study participants by country income groups

COVID-symptoms and vital signs	Overall, N (%)	HIC N (%)	UMIC N (%)	LMIC N (%)	LIC N (%)	p-value for difference
Diagnosed by using RT-PCR	5050 (95.0%)	1350 (98.8%)	371 (98.7%)	2399 (91.1%)	930 (99.3%)	<0.001
Median time from symptom onset to admission (IQR) in minutes	5 (3-8)	21.5 (489.5)	26.8 (378.4)	6.5 (15.8)	27.1 (572.7)	0.4
H/o self-reported fever	3526 (66.4%)	933 (68.3%)	157 (41.8%)	1814 (68.9%)	622 (66.4%)	<0.001
Cough	3624 (68.2%)	993 (72.6%)	253 (67.3%)	1726 (65.6%)	652 (69.6%)	<0.001
Dyspnoea OR Tachypnoea	3308 (62.3%)	703 (51.4%)	235 (62.5%)	1699 (64.5%)	671 (71.6%)	<0.001
Heart rate (beats/min), mean (SD)	92.1 (17.8)	88.2 (14.2)	95.1 (18.4)	92.6 (17.9)	95.5 (21.1)	<0.001
Systolic BP (mmHg), mean (SD)	128.8 (20.9)	128.6 (18.7)	132.3 (19.5)	128.0 (21.1)	130.0 (23.4)	<0.001
Diastolic BP (mmHg), mean (SD)	78.2 (13.0)	78.4 (12.1)	82.2 (14.6)	78.0 (12.3)	76.9 (15.1)	<0.001
Shortness of Breath (SOB)						<0.001
No	1341 (25.2%)	514 (37.6%)	147 (39.1%)	495 (18.8%)	185 (19.7%)	
SOB < 100m	1335 (25.1%)	238 (17.4%)	185 (49.2%)	466 (17.7%)	447 (47.7%)	
SOB 100- 500m	479 (9.0%)	65 (4.8%)	23 (6.1%)	365 (13.9%)	26 (2.8%)	
SOB > 500m	225 (4.2%)	19 (1.4%)	3 (0.8%)	199 (7.6%)	4 (0.4%)	
**Co-morbidities (Cardiovascular)**						
Hypertension	2511 (47.3%)	680 (49.7%)	160 (42.6%)	1253 (47.6%)	418 (44.6%)	<0.001
Diabetes	1700 (32.0%)	301 (22.0%)	77 (20.5%)	1025 (38.9%)	297 (31.7%)	<0.001
Coronary artery disease	580 (10.9%)	151 (11.0%)	13 (3.5%)	331 (12.6%)	56 (6.0%)	<0.001
Heart Failure	290 (5.5%)	125 (9.1%)	12 (3.2%)	97 (3.7%)	29 (3.1%)	<0.001
Stroke	197 (3.7%)	85 (6.2%)	13 (3.5%)	70 (2.7%)	34 (3.6%)	<0.001
Atrial Fibrillation	159 (3.0%)	100 (7.3%)	4 (1.1%)	21 (0.8%)	29 (3.1%)	<0.001
Peripheral vascular disease	106 (2.0%)	61 (4.5%)	3 (0.8%)	13 (0.5%)	13 (1.4%)	<0.001
Cardiomyopathies	60 (1.1%)	21 (1.5%)	5 (1.3%)	21 (0.8%)	2 (0.2%)	<0.001
Rheumatic Heart Disease	56 (1.1%)	32 (2.3%)	0 (0.0%)	22 (0.8%)	6 (0.6%)	<0.001
Chagas disease	36 (0.7%)	28 (2.0%)	0 (0.0%)	2 (0.1%)	106 (11.3%)	<0.001
Congenital heart disease	182 (3.4%)	37 (2.7%)	0 (0.0%)	39 (1.5%)	24 (2.6%)	<0.001
Valvular disease	118 (2.2%)	38 (2.8%)	3 (0.8%)	53 (2.0%)	80 (8.5%)	<0.001
**Co-morbidities (Non-Cardiovascular)**						
Chronic kidney disease	404 (7.6%)	135 (9.9%)	14 (3.7%)	175 (6.6%)	66 (7.0%)	<0.001
Chronic pulmonary disease	208 (3.9%)	69 (5.0%)	3 (0.8%)	71 (2.7%)	30 (3.2%)	<0.001
Asthma	219 (4.1%)	54 (4.0%)	22 (5.9%)	113 (4.3%)	42 (4.5%)	<0.001
Chronic Immunosuppression	136 (2.6%)	67 (4.9%)	10 (2.7%)	17 (0.6%)	13 (1.4%)	<0.001
HIV	71 (1.3%)	8 (0.6%)	44 (11.7%)	6 (0.2%)	13 (1.4%)	<0.001
Tuberculosis	56 (1.1%)	7 (0.5%)	22 (5.9%)	14 (0.5%)	34 (3.6%)	<0.001
Cancer on chemotherapy	114 (2.1%)	56 (4.1%)	1 (0.3%)	23 (0.9%)	24 (2.6%)	<0.001
Renal replacement therapy	62 (1.2%)	13 (1.0%)	3 (0.8%)	22 (0.8%)	16 (1.7%)	<0.001
Previous organ transplant	45 (0.8%)	21 (1.5%)	1 (0.3%)	7 (0.3%)	14 (1.5%)	<0.001

HIC=high income countries; UMIC=upper middle-income countries; LMIC=lower middle-income countries; LIC=low-income countries

eTable 2. Pre-admission medications in the study population

Pre-admission medications (Cardiovascular)	Overall N (%)	Survivors N (%)	In-hospital deaths N (%)	Post discharge 30-day deaths N (%)	p-value for difference
Beta-blockers	897 (16.9)	760 (16.8)	115 (16.8)	22 (18.6)	0.44
Alpha-blockers	131 (2.5)	108 (2.4)	17 (2.5)	6 (5.1)	0.19
Diuretics oral	625 (11.8)	512 (11.3)	100 (14.6)	13 (11.0)	0.23
RAAS inhibitors	1416 (26.7)	1183 (26.2)	204 (29.9)	29 (24.6)	0.013
Anti-coagulant/anti-platelets	972 (18.3)	780 (17.3)	161 (23.6)	31 (26.3)	0.002
Anti-arrhythmic drugs	72 (1.4)	59 (1.3)	10 (1.5)	3 (2.5)	0.57
Calcium antagonists	921 (17.3)	774 (17.2)	129 (18.9)	18 (15.3)	0.77
Nitrates	161 (3.0)	122 (2.7)	32 (4.7)	7 (5.9)	0.019
Statins	873 (16.4)	725 (16.1)	122 (17.9)	26 (22.0)	0.24
Aldosterone antagonists	114 (2.1)	82 (1.8)	27 (4.0)	5 (4.2)	0.004
Endocarditis prophylaxis	8 (0.2)	5 (0.1)	3 (0.4)	0 (0.0)	0.17
**Pre-admission medications (non-Cardiovascular)**					
Oral hypoglycemic agents	915 (17.2)	754 (16.7)	148 (21.7)	13 (11.0)	0.002
Insulin	649 (12.2)	504 (11.2)	129 (18.9)	16 (13.6)	0.045
NSAIDs (regular use)	186 (3.5)	174 (3.9)	9 (1.3)	3 (2.5)	<0.001
Anti-depressants	226 (4.3)	202 (4.5)	21 (3.1)	3 (2.5)	0.023
Blood transfusion (in last 2 weeks)	29 (0.5)	23 (0.5)	3 (0.4)	3 (2.5)	0.005
Antiretroviral therapy	79 (1.5)	69 (1.5)	7 (1.0)	3 (2.5)	0.17
Influenza vaccine in the past 6 month	119 (2.2)	106 (2.3)	10 (1.5)	3 (2.5)	0.31

RAAS= Renin-angiotensin-aldosterone system

eTable 3. Supportive care, and medications during hospitalization

**Supportive care during hospitalization**	**Overall, N (%)**	**Survivors N (%)**	**In-hospital deaths, N (%)**	**Post discharge 30-day deaths, N (%)**	**p-value for difference**
Oxygen therapy	3384 (63.7)	2711 (60.1)	584 (85.5)	89 (75.4)	<0.001
Oxygen saturation done	2906 (54.7)	2374 (52.6)	453 (66.3)	79 (66.9)	<0.001
SpO2 level (), mean (SD)	2836	93.7 (7.3)	89.3 (10.2)	93.0 (6.5)	<0.001
Non-invasive ventilation (e.g., BIPAP/CPAP)	671 (12.6)	492 (10.9)	159 (23.3)	20 (16.9)	<0.001
Invasive ventilation (Any)	395 (7.4)	177 (3.9)	205 (30.0)	13 (11.0)	<0.001
Inotropes/vasopressors	361 (6.8)	158 (3.5)	185 (27.1)	18 (15.3)	<0.001
Extracorporeal (ECMO) support	15 (0.3)	8 (0.2)	7 (1.0)	0 (0.0)	<0.001
Prone position	1339 (25.2)	1051 (23.3)	269 (39.4)	19 (16.1)	<0.001
**Medications during hospitalization**					
Intravenous fluids	2965 (55.8)	2433 (53.9)	459 (67.2)	73 (61.9)	<0.001
Antiviral	1723 (32.4)	1434 (31.8)	239 (35.0)	50 (42.4)	0.020
Corticosteroid	3690 (69.5)	3011 (66.7)	594 (87.0)	85 (72.0)	<0.001
Antibiotic	3617 (68.1)	2919 (64.7)	594 (87.0)	104 (88.1)	<0.001
Antifungal agent	174 (3.3)	124 (2.7)	41 (6.0)	9 (7.6)	<0.001
Antimalarial agent	220 (4.1)	196 (4.3)	24 (3.5)	0 (0.0)	0.048
Non-steroidal anti-inflammatory drugs (NSAID)	726 (13.7)	631 (14.0)	79 (11.6)	16 (13.6)	<0.001
RAAS inhibitors	1226 (23.1)	1034 (22.9)	162 (23.7)	30 (25.4)	<0.001

SD=standard deviation; SpO2= Oxygen saturation; BIPAP= bi-level positive airway pressure; CPAP= Continuous Positive Airway Pressure

eTable 4. Overall status at discharge and 30-day follow-up

Status at discharge	Overall, N (%)
Discharged	4072 (76.6)
Alive, and still an inpatient	164 (3.1)
Transfer to other facility	228 (4.3)
Death	683 (12.9)
Palliative discharge	3 (0.1)
**30 Day Outcome**	
Re-Hospitalized	72 (1.6)
Death post-discharge	118 (2.6)
**Overall death**	801 (15.1)
**Causes of death**	
Respiratory failure	317 (39.3)
Sudden cardiac death	153 (20.0)
Other cardiovascular	140 (17.4)
Other	174 (21.6)
**Ability to self-care at discharge versus before illness**	
Same as before illness	3731 (70.2)
Worse	204 (3.8)
Unknown	137 (2.6)
**Fully recovered from COVID-19 at 30-day (N=4512)**	
Strongly disagree	78 (2.3)
Disagree	236 (6.9)
Slightly disagree	251 (7.4)
Slightly agree	830 (24.4)
Agree	1642 (48.2)
Strongly agree	370 (10.9)

eTable 5. Factors associated with in-hospital death and post-discharge 30-day death in COVID-19 hospitalized patients

	Unadjusted		[1] Adjusted for demographics		[2] Adjusted for demographics & clinical	
	In-hospital death RR (95% CI)	30-day death RR (95% CI)	In-hospital death RR (95% CI)	30-day death RR (95% CI)	In-hospital death RR (95% CI)	30-day death RR (95% CI)
**Age (years)**						
<45 (Ref)	1.0	1.0	1.0	1.0	1.0	1.0
46-60	1.98 (1.47, 2.66)	2.46 (1.13, 5.34)	2.01 (1.49, 2.7)	2.43 (1.12, 5.28)	1.84 (1.36, 2.49)	2.42 (1.11, 5.30)
≥60	4.11 (3.17, 5.34)	5.86 (2.94, 11.69)	4.53 (3.48, 5.9)	6.16 (3.08, 12.33)	4.02 (3.07, 5.27)	6.17 (3.05, 12.50)
**Sex**						
Male	1.21 (1.03, 1.43)	1.90 (1.26, 2.87)	1.19 (1.01, 1.42)	1.85 (1.23, 2.80)	1.19 (1, 1.42)	1.85 (1.22, 2.80)
**Ethnicity**						
Caucasian	1.0	1.0	1.0	1.0	1.0	1.0
Hispanic	5.53 (3.87, 7.92)	1.55 (0.47, 5.11)	6.05 (4.20, 8.70)	1.68 (0.51, 5.54)	5.83 (4.05, 8.40)	1.65 (0.50, 5.45)
Black	2.91 (2.03, 4.17)	1.87 (0.67, 5.16)	3.62 (2.51, 5.21)	2.36 (0.85, 6.57)	3.56 (2.47, 5.13)	2.29 (0.83, 6.37)
Middle Eastern	1.06 (0.60, 1.86)	6.18 (2.35, 16.23)	1.04 (0.59, 1.83)	5.93 (2.25, 15.65)	1.01 (0.57, 1.79)	5.95 (2.25, 15.69)
Asian	2.64 (1.91, 3.64)	4.39 (1.90, 10.15)	3.07 (2.21, 4.25)	5.09 (2.2, 11.79)	2.86 (2.06, 3.98)	4.93 (2.12, 11.45)
Other	2.47 (1.60, 3.82)	4.53 (1.66, 12.36)	2.64 (1.70, 4.09)	4.86 (1.78, 13.31)	2.53 (1.63, 3.93)	4.86 (1.77, 13.31)
**Region**						
Europe	1.0	1.0	1.0	1.0	1.0	1.0
Asia Pacific	0.36 (0.17, 0.74)	-	0.44 (0.21, 0.90)	-	0.44 (0.21, 0.91)	-
Latin America	4.88 (3.45, 6.92)	3.60 (1.36, 9.54)	4.99 (3.50, 7.09)	3.56 (1.34, 9.44)	4.84 (3.4, 6.89)	3.58 (1.35, 9.51)
Middle East	1.02 (0.56, 1.85)	6.91 (2.6, 18.36)	0.97 (0.53, 1.77)	6.43 (2.41, 17.15)	0.96 (0.52, 1.75)	6.5 (2.44, 17.34)
North America	0.39 (0.05, 2.91)	6.16 (1.21, 31.32)	0.37 (0.05, 2.75)	5.49 (1.07, 28.21)	0.34 (0.05, 2.56)	5.9 (1.14, 30.53)
Southeast Asia	3.67 (2.67, 5.04)	6.74 (2.92, 15.57)	4.12 (2.98, 5.68)	7.40 (3.2, 17.13)	3.88 (2.81, 5.37)	7.4 (3.18, 17.21)
Sub Saharan Africa	3.86 (2.75, 5.42)	2.05 (0.76, 5.58)	4.65 (3.3, 6.56)	2.48 (0.91, 6.76)	4.57 (3.24, 6.45)	2.44 (0.89, 6.65)
**Income group**						
HIC	1.0	1.0	1.0	1.0	1.0	1.0
LIC	2.55 (1.67, 3.88)	2.61 (0.92, 7.4)	2.96 (1.93, 4.54)	3.03 (1.07, 8.63)	2.95 (1.92, 4.52)	2.98 (1.05, 8.49)
LMIC	3.91 (2.95, 5.18)	6.15 (3.08, 12.25)	4.03 (3.03, 5.35)	6.16 (3.08, 12.3)	3.82 (2.87, 5.08)	6.07 (3.03, 12.17)
UMIC	5.58 (4.13, 7.53)	2.64 (1.15, 6.07)	5.51 (4.07, 7.46)	2.49 (1.08, 5.74)	5.34 (3.94, 7.24)	2.49 (1.08, 5.74)
**Smoking status**						
Never (Ref)	1.0	1.0	1.0	1.0	1.0	1.0
Current smoker	0.48 (0.31, 0.74)	0.97 (0.44, 2.15)	0.59 (0.37, 0.95)	0.98 (0.43, 2.22)	0.61 (0.38, 0.97)	1.01 (0.44, 2.31)
Former smoker	1.02 (0.80, 1.31)	1.25 (0.72, 2.17)	0.86 (0.66, 1.13)	0.94 (0.52, 1.70)	0.85 (0.65, 1.11)	0.97 (0.54, 1.74)
Unknown	1.83 (1.52, 2.2)	2.10 (1.38, 3.19)	1.69 (1.37, 2.07)	1.79 (1.12, 2.84)	1.66 (1.35, 2.04)	1.77 (1.11, 2.82)

**Pre-existing chronic conditions**						
Hypertension	1.62 (1.37, 1.90)	0.94 (0.65, 1.36)	1.20 (1.00, 1.43)	0.71 (0.48, 1.04)	1.10 (0.91, 1.32)	0.68 (0.46, 1.01)
Coronary heart disease	1.59 (1.26, 2.01)	3.19 (2.10, 4.87)	1.38 (1.08, 1.76)	2.13 (1.37, 3.31)	1.30 (1.01, 1.66)	2.17 (1.39, 3.40)
Stroke	1.17 (0.78, 1.76)	2.53 (1.30, 4.94)	1.03 (0.68, 1.58)	1.80 (0.91, 3.58)	1.01 (0.66, 1.54)	1.84 (0.92, 3.66)
Heart failure	1.25 (0.90, 1.73)	1.11 (0.51, 2.42)	1.21 (0.86, 1.71)	1.15 (0.52, 2.55)	1.16 (0.82, 1.65)	1.15 (0.52, 2.54)
Renal disease	2.03 (1.57, 2.62)	2.70 (1.63, 4.48)	1.88 (1.43, 2.45)	2.62 (1.55, 4.42)	1.75 (1.33, 2.29)	2.61 (1.54, 4.44)
COPD/Asthma	1.10 (0.82, 1.47)	0.65 (0.28, 1.48)	1.00 (0.74, 1.36)	0.55 (0.24, 1.27)	1.01 (0.75, 1.37)	0.55 (0.24, 1.27)
Diabetes	1.91 (1.62, 2.25)	1.61 (1.11, 2.34)	1.45 (1.22, 1.73)	1.09 (0.74, 1.60)	1.45 (1.22, 1.73)	1.08 (0.73, 1.59)
Tuberculosis	0.94 (0.42, 2.08)	-	0.86 (0.38, 1.95)	-	0.89 (0.39, 2.03)	-
HIV	0.64 (0.27, 1.48)	1.87 (0.58, 6.05)	0.78 (0.32, 1.88)	5.55 (1.49, 20.68)	0.81 (0.34, 1.95)	5.66 (1.52, 21.13)
Oxygen therapy	3.98 (3.18, 4.98)	2.01 (1.31, 3.06)	3.57 (2.84, 4.5)	1.81 (1.17, 2.81)	3.48 (2.76, 4.39)	1.80 (1.16, 2.80)
ICU admission	4.75 (4.01, 5.63)	3.42 (2.36, 4.94)	4.15 (3.44, 5.01)	3.41 (2.31, 5.04)	4.09 (3.39, 4.94)	3.37 (2.28, 4.97)
Respiratory rate, mean (SD)	1.11 (1.09, 1.12)	1.06 (1.02, 1.09)	1.1 (1.08, 1.12)	1.07 (1.03, 1.12)	1.1 (1.08, 1.12)	1.07 (1.03, 1.12)
**Medications**						
Beta-blockers	0.87 (0.69, 1.11)	1.12 (0.65, 1.92)	0.99 (0.79, 1.24)	0.95 (0.59, 1.54)	0.93 (0.74, 1.17)	0.94 (0.58, 1.52)
Diuretics oral	1.26 (0.98, 1.62)	0.95 (0.51, 1.78)	1.28 (1, 1.63)	0.96 (0.53, 1.75)	1.23 (0.96, 1.57)	0.96 (0.52, 1.74)
ACE - inhibitors	0.72 (0.54, 0.96)	0.45 (0.2, 1.01)	0.85 (0.63, 1.13)	0.54 (0.24, 1.19)	0.79 (0.59, 1.06)	0.53 (0.24, 1.18)
Anti-coagulant/anti-platelets/NOACs drugs	1.36 (1.08, 1.71)	1.98 (1.16, 3.38)	1.33 (1.08, 1.63)	1.14 (0.74, 1.76)	1.25 (1.01, 1.54)	1.14 (0.73, 1.77)
Angiotensin II receptor blockers	1.24 (0.98, 1.57)	1.36 (0.8, 2.33)	1.01 (0.82, 1.25)	1.00 (0.62, 1.59)	0.97 (0.78, 1.2)	1.00 (0.63, 1.60)
Statins	0.96 (0.76, 1.22)	1.56 (0.91, 2.65)	0.9 (0.72, 1.12)	0.97 (0.61, 1.52)	0.82 (0.66, 1.03)	0.94 (0.59, 1.50)
Anti-diabetic drugs (Oral hypoglycemic agents)	0.67 (0.49, 0.93)	0.34 (0.16, 0.77)	1.06 (0.86, 1.3)	0.43 (0.24, 0.78)	0.70 (0.54, 0.9)	0.28 (0.15, 0.53)
NSAIDs (regular use)	0.26 (0.13, 0.52)	0.68 (0.2, 2.23)	0.33 (0.17, 0.66)	0.60 (0.18, 1.94)	0.34 (0.17, 0.67)	0.60 (0.19, 1.96)
ACE or ARB	0.99 (0.79, 1.24)	0.77 (0.45, 1.3)	0.93 (0.73, 1.19)	0.89 (0.52, 1.54)	0.92 (0.72, 1.17)	0.90 (0.52, 1.56)
**BMI**						
18.0-24.9	1.0	1.0	1.0	1.0		
<18.0	0.65 (0.26, 1.65)	0.77 (0.1, 5.75)	1.00 (0.38, 2.61)	1.26 (0.16, 9.96)	0.97 (0.37, 2.54)	1.20 (0.15, 9.5)
25.0-29.9	1.04 (0.81, 1.32)	0.53 (0.26, 1.05)	1.01 (0.78, 1.31)	0.52 (0.26, 1.05)	0.99 (0.76, 1.28)	0.51 (0.25, 1.04)
≥30	1.02 (0.77, 1.35)	0.68 (0.33, 1.43)	1.08 (0.79, 1.48)	0.81 (0.37, 1.79)	1.01 (0.74, 1.39)	0.77 (0.35, 1.70)

SD=standard deviation; BMI=body mass index; CI=confidence interval; ACE=angiotensin converting enzyme; ARB=angiotensin receptor blocker; NOAC=novel oral anticoagulants; HIV=human immunodeficiency virus; HIC=high income countries; UMIC=upper middle-income countries; LMIC=lower middle-income countries; LIC=low-income countries

Adjusted risk[1] (Model 1): adjusted for demographic variables (age, sex, ethnicity)
Adjusted risk[2] (Model 2): adjusted for demographic and clinical characteristics (Diabetes, COPD/asthma)
