# Supplementary material for: Cardiovascular Risk Factors and Clinical Outcomes among Patients Hospitalized with COVID-19: Findings from the World Heart Federation COVID-19 Study
Source: Glob Heart. 2022 Jun 15;17(1):40. doi: 10.5334/gh.1128 (PMC9205371; doi:10.5334/gh.1128)
Supplement: Appendix 1. — Site wise patient recruitment in the WHF COVID-19 Study. [file gh-17-1-1128-s2.pdf]

Appendix 1. Site wise patient recruitment in the WHF COVID-19 Study.

Study Site Name	Patients enrolled
Dayanand Medical College, Punjab, India	501
NHO Tochigi MC, Japan	412
Amin and Khorshid Hospital, Iran	300
Kuwait Bangladesh Friendship Govt. Hospital, Bangladesh	300
Bangladesh Specialized Hospital, Bangladesh	300
Dhaka Medical College, Bangladesh	300
LEVY Mwanawasa, Zambia	200
FVDL, Colombia	200
Groote Schuur Hospital, South Africa	189
OOUTH, Nigeria	181
AIIMS Jodhpur, India	171
Kazan 7 Emergency Medicine Center, Russia	161
Tomsk National Research Medical Centre, Russia	146
Almazov National Medical Research Centre, Russia	145
ISSSTE Clinica Hospital, Guanajuato	135
University College Hospital Ibadan, Nigeria	122
University Clinical Centre of the Republic of Srpska, Bosnia	122
INDC, Brazil	121
USZ, Switzerland	119
National Cardiovascular Center Harapan Kita, Indonesia	107
Sanatorio Güemes Hospital, Argentina	105
Tabba Heart Institute, Pakistan	101
The Mombasa Hospital, Kenya	94
CHULN-HSM, Portugal	91
KATH, Ghana	79
AIIMS New Delhi, India	67
RUDN University, City Clinical Hospital, Russia	64
CGTRH-Mombasa, Kenya	57
Apollo Hospital, Hyderabad, India	53
Emory University, Atlanta	50
Apollo Medical College, Jubilee Hills	50
Ryazan State Medical University, Russia	50
Hospital Dr.Hernán Henriquez Aravena, Chile	47
Kyoto Medical Center, Japan	44
Hospital de Clinicas of the University, Argentina	40
Fedail Hospital, Sudan	25
Clinica de Occidente, Colombia	25
TSMU, Georgia	23
Kitasato University, Japan	15
LUTH, Nigeria	1
**Grand Total**	**5313**
